# A metabarcode based (species) inventory of the northern Adriatic phytoplankton

**DOI:** 10.3897/BDJ.11.e106947

**Published:** 2023-09-25

**Authors:** Lana Grižančić, Ana Baričević, Mirta Smodlaka Tanković, Ivan Vlašiček, Mia Knjaz, Ivan Podolšak, Tjaša Kogovšek, Martin Andreas Pfannkuchen, Daniela Marić Pfannkuchen

**Affiliations:** 1 Ruder Boskovic Institute, Centre for Marine Research, Rovinj-Rovigno, Croatia Ruder Boskovic Institute, Centre for Marine Research Rovinj-Rovigno Croatia

**Keywords:** metabarcode, HTS, 18S, phytoplankton, northern Adriatic, diatoms, dinoflagellates

## Abstract

**Background:**

The northern Adriatic is characterised as the coldest and most productive marine area of the Mediterranean, which is due to high nutrient levels introduced by river discharges, the largest of which is the Italian Po River (at the same time also the largest freshwater input into the Mediterranean). The northern Adriatic is a very shallow marine ecosystem with ocean current patterns that result in long retention times of plankton in the area. The northern Adriatic phytoplankton biodiversity and abundance are well-studied, through many scientific and long-term monitoring reports. These datasets were based on phytoplankton morphological traits traditionally obtained with light microscopy. The most recent comprehensive eastern Adriatic phytoplankton checklist was published more than 20 years ago and is still valuable today. Since phytoplankton taxonomy and systematics are constantly being reviewed (partly also due to new molecular methods of species identification that complement classical methodologies), checklists need to be updated and complemented. Today, metabarcoding of molecular markers gains more and more importance in biodiversity research and monitoring. Here, we report the use of high throughput sequencing methods to re-examine taxonomic richness and provide updated knowledge of phytoplankton diversity in the eastern northern Adriatic to complement the standardised light microscopy method.

**New information:**

This study aimed to report an up-to-date list of the phytoplankton taxonomic richness and phylogenetic relationships in the eastern northern Adriatic, based on sequence variability of barcoding genes resolved with advanced molecular tools, namely metabarcoding. Here, metabarcoding is used to complement standardised light microscopy to advance conventional monitoring and research of phytoplankton communities for the purpose of assessing biodiversity and the status of the marine environments. Monthly two-year net sampling targeted six phytoplankton groups including Bacillariophyceae (diatoms) and Chrysophyceae (golden algae) belonging to Ochrophyta, Dinophyceae (dinoflagellates), Cryptophyceae (cryptophytes), Haptophyta (mostly coccolithophorids) and Chlorophyta with Prasinophyceae (prasinophytes) and Chlorophyceae (protist green algae). Generated sequence data were taxonomically assigned and redistributed in two kingdoms, five classes, 32 orders, 49 families and 67 genera. The most diverse group were dinoflagellates, comprising of 34 found genera (48.3%), following by diatoms with 23 (35.4%) and coccolithophorids with three genera (4.0%). In terms of genetic diversity, results were a bit different: a great majority of sequences with one nucleotide tolerance (ASVs, Amplicon sequence variants) assigned to species or genus level were dinoflagellates (83.8%), 13.7% diatoms and 1.6% Chlorophyta, respectively. Although many taxa have not been detected that have been considered as common in this area, metabarcoding revealed five diatoms and 20 dinoflagellate genera that were not reported in previous checklists, along with a few species from other targeted groups that have been reported previously. We here describe the first comprehensive 18S metabarcode inventory for the northern Adriatic Sea.

## Introduction

The entire marine community wealth is directly linked to phytoplankton richness and abundance. Their relatively high turnover rate results in rapid response to biotic and abiotic environmental changes making phytoplankton a valuable indicator for monitoring assessments (Goodwin et al. 2017, Porter and Hajibabaei 2018). Defining phytoplankton community structure in marine environments and their spatial and temporal changes are, therefore, included in the Marine Strategy Framework Directive (MSFD) as indicators for Good Environmental Status assessments (2008/56/EC).

Defining environmental status of the aquatic environment assessing phytoplankton composition and biomass is of great importance, especially in dynamic coastal waters such as the northern Adriatic (NA). Located in the northernmost area of the Mediterranean, the northern Adriatic is a shallow basin, with depths up to 50 m and regular exchange of water with the Mediterranean Sea (Gacic et al. 2010) through dominant cyclonic circulation along the eastern Adriatic coast (EAC) bringing high salinity oligotrophic water into the northern Adriatic (Franco and Michelato 1992). Its thermohaline properties are mostly seasonal, mainly shaped by: (1) cold and dry inland bora wind in winter (Belušić et al. 2013) triggering cold dense water formation (Mihanovic et al. 2013) with strong vertical mixing, resulting in stratification absence and (2) large river discharges (Raicich 1996), mostly by the River Po that shape both the salinity and temperature, maintaining strong stratification during the warm part of a year with low-salinity nutrient-rich water spreading horizontally extending anticyclonally into the northern Adriatic interior (Giani et al. 2012). As a result of river intake, a large amount of inorganic, as well as organic nutrients are introduced (Degobbis et al. 2000, Cozzi et al. 2018, affecting circulation by forming southward highly eutrophic West Adriatic Current (WAC), transferring nutrients along the western Adriatic coast (Zavatarelli et al. 1998). Consequently, the northern Adriatic is characterised as one of the most productive areas of the Mediterranean (D’Ortenzio and D’Alcalà 2009) with diverse physical and chemical gradients that affect seasonal variations of phytoplankton species diversity in this area.

There are many long-term studies reporting phytoplankton diversity and abundance as key indicators for changes occurring in the eastern northern Adriatic (Cabrini et al. 2012, Maric et al. 2012, Godrijan et al. 2013, Mozetič et al. 2019, France et al. 2021). Described phytoplankton taxonomic composition is based on morphological traits and, therefore, some species playing important ecological roles may be overlooked in biodiversity surveys. Many papers report phytoplankton diversity and composition acquired with classical microscopy (Revelante and Gilmartin 1976, Bosak et al. 2009, Fanuko and Valcic 2009, Vilicic et al. 2009, Mozetič et al. 2019). However, the most comprehensive recent phytoplankton checklist for the eastern Adriatic Sea phytoplankton remains (Vilicic et al. 2002) which we also use as a reference for biodiversity recovered by light microscopy in this study.

Identification of multiple species from a bulk sample containing entire organisms or from a single environmental sample can be termed DNA metabarcoding (Taberlet et al. 2012). Recent technological developments in molecular ecology using genetic approaches have expanded our capacity to describe marine plankton community diversity (Caron et al. 2011, Leray and Knowlton 2017) and allow us to better understand phylogenetic relationships and taxonomic structures in environmental samples. Next generation high-throughput sequencing technologies have become a common research tool for biodiversity evaluation with the power to detect even the rarest members of a specific community (Sogin et al. 2006), as well as discriminate between closely-related and cryptic species, based on sequence similarity. The results provide complementary rather than identical phytoplankton community structure estimates when compared to conventional approaches (Keck et al. 2022). Studies of phytoplankton community composition in the northern Adriatic adopting metabarcoding were previously conducted on the eastern Italian coast for seven stations and dates dates inside and in front of the lagoon of Venice (Armeli Minicante et al. 2019). Here, we report a phytoplankton species inventory acquired from metabarcoding data at two long-term monitoring stations near Rovinj (Croatia) collected monthly over the course of 2 years. This detailed phytoplankton genetic diversity information represents an important molecular tool to analyse community structure, track seasonal dynamics and overall, to better understand future metabarcoding studies occurring in the eastern northern Adriatic and going towards metabarcoding biodiversity monitoring.

## Materials and methods

### Sampling description

**Figure 1.**
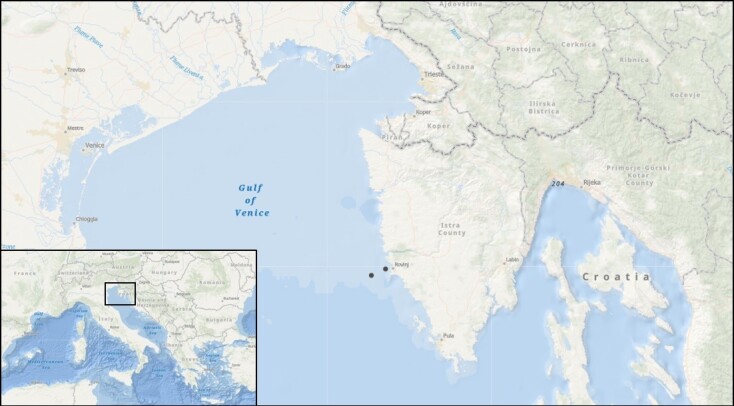
Map of two sampling sites located near Rovinj with long sampling history used as reference points for sample collection.

Sampling took place at two stations with long term sampling history and a continuous monitoring programme coordinated by the Centre for Marine Research (CIM), monthly during the years 2020 and 2021. Stations RV001 and RV004 (45°4'48''N, 13°36'36''E and 45°3'42.66''N, 13°32'56.976''E) are located 1 and 4 nautical miles off the Croatian coast and are used in routine monitoring for the collection of representative phytoplankton samples for the northern Adriatic Sea (Fig. 1). Samples for metabarcoding analysis were collected at each site using a phytoplankton net with a defined mesh size of 50 µm (Hydrobios, Germany). Samples were collected by vertical net hauls from ∼ 30 m depth to the surface to cover species from the entire water column. Concentrated samples were collected in 500 ml plastic bottles and filtered on 1.2 μm cellulose ester membranes (47 mm, Whatman, UK) until complete filter saturation (total volume ranging from 20 ml up to 150 ml). Filters were stored in small Petri dishes, at -80°C as suggested by Baricevic et al. (2022) until further lab processing.

### Laboratory protocol

Total DNA was extracted from stored filters using either Gentra Puregene DNA Isolation Kit (Qiagen, Germany) or NucleoSpin eDNA Water kit (Macherey-Nagel, Germany), both according to the manufacturer′s protocol. Isolated genomic DNA was eluted in 100 µl per sample and obtained concentrations were quantified by measuring DNA concentration/absorbance spectra using the Nanophotometer spectrophotometer (Implen, Germany). Samples were stored at 4°C until further processing. DNA metabarcoding library preparation and sequencing were carried out by AllGenetics & Biology SL (www.allgenetics.eu). DNA concentration was quantified for each extract using the Qubit High Sensitivity dsDNA Assay (Thermo Fisher Scientific). For DNA metabarcoding library preparation, eukaryote specific primers TAReuk454FWD1 (5′-CCAGCA(G/C)C(C/T)GCGGTAATTCC-3′) and TAReukREV3 (5′-ACTTTCGTTCTTGAT(C/T)(A/G)A-3′) (Stoeck et al. 2010, Piredda et al. 2017) were used, targeting around 430 bp of the V4 hypervariable region of the small subunit ribosomal RNA gene with the Illumina sequencing primer sequences attached at their 5’ ends. In the amplification step, PCRs were conducted in a final volume of 12.5 μl, containing 1.25 μl of template DNA optimised by diluting the starting template DNA, 0.5 μM of the primers, 3.13 μl of Supreme NZYTaq 2x Green Master Mix (NZYTech) and ultrapure water up to 12.5 μl. The reaction mixture was incubated as follows: an initial denaturation step at 95°C for 5 min, followed by 35 cycles of 95°C for 30 s, 48°C for 45 s, 72°C for 45 s and a final extension step at 72°C for 7 min. The oligonucleotide indices were attached in a second amplification step with identical conditions, but only 5 cycles and 60ºC as the annealing temperature (Vierna et al. 2017). The library size was verified by running the libraries on 2% agarose gels stained with GreenSafe (NZYTech) and imaging them under UV light. Then, the libraries were purified using the Mag-Bind RXNPure Plus magnetic beads (Omega Biotek), following the instructions provided by the manufacturer. Finished libraries were pooled in equimolar amounts according to the results of a Qubit dsDNA HS Assay (Thermo Fisher Scientific) quantification. These pools also contained a testimonial amount (1 μl) of the PCR negative controls. The pool was sequenced in a fraction of NovaSeq PE250 (Illumina) aiming for a total output of 4 gigabases. Illumina paired-end raw data consisted of forward (R1) and reverse (R2) reads and were stored separately sorted by library, as fastq files.

### Custom Reference database (CIMPhy18) preparation

The CIM Phytoplankton 18S ribosomal RNA reference database (CIMPhy18) was assembled using ARB software v.7.0 (Ludwig et al. 2004) by merging downloaded phytoplankton sequence records from the Silva (Quast et al. 2013) and Protist Ribosomal Reference (PR2) (Guillou et al. 2013) databases. Phytoplankton groups included Dinophyceae (dinoflagellates), Ochrophyta containing Bacillariophyceae (diatoms) and Chrysophyceae (golden algae), Cryptophyceae (cryptophytes), Haptophyta (mostly coccolithophorids) and Chlorophyceae (green micro algae). First, the Silva-aligned database was imported into ARB software with all additional data and NCBI accession numbers linked to phytoplankton taxa were compared between Silva and PR2 databases. PR2 accession number entries that were not present in the Silva database were downloaded from Entrez Molecular Sequence Database System (www.ncbi.nlm.nih.gov/Web/Search/entrezfs.html) in genbank (.gb) format and imported into ARB. Phytoplankton groups/phyla chosen for the reference database assembly were aligned in ARB Sequence Editor and a reference database phylogenetic tree was constructed. Relevant in-house sequences from the in-house CIM phytoplankton culture collection, representing barcoded sequences from northern Adriatic common phytoplankton taxa, were used to extend the database and to include species not included in the standard release of the databases. To acquire information about the taxonomic completeness of the constructed database, phytoplankton taxa entries were compared to: (1) the eastern Adriatic phytoplankton checklist (Vilicic et al. 2002) and (2) the list of northern Adriatic phytoplankton species from long-term monitoring data. Both lists were obtained from long-term studies with light microscopy. Before comparison, taxonomic data were normalised using the Species matching tool from GBIF (www.gbif.org) to ensure accurate classification and taxonomic uniformity. Sequences for missing taxa were searched in NCBI (www.ncbi.nlm.nih.gov), curated and, if suitable, added to the CIMPhy18 database along with accompanying metadata. Sequences for the reference database were extracted in fasta format and their accompanying taxonomy in a tax file, prepared to be imported into Mothur (Schloss et al. 2009).

### Bioinformatic pipeline

Illumina paired-end reads for the small subunit region were processed using a custom script for the Mothur pipeline v.1.47.0 (Schloss et al. 2009), based on MiSeq standard operating procedure (https://mothur.org/wiki/miseq_sop/). Contigs were assembled from fastq files containing forward and reverse Illumina MiSeq reads and trimmed to the overlapping section, with no ambiguous bases allowed; the maximum homopolymer size was 8 bp and the maximum tolerated sequence length 450 bp. Sequences were screened for chimeras using the VSEARCH command (Rognes et al. 2016), removing sequences with chimeras from analysis from their group only (dereplicate=t). Clustered sequences are reported as amplicon sequence variants, ASVs (Callahan et al. 2017), with one nucleotide tolerance (cutoff = 1) eliminating erroneous sequences formed due to sequencing mistakes and operational taxonomic units, OTUs were assembled, based on fixed sequence 97%- and 99%-identity (99%-OTUs and 97%-OTUs) threshold. Both times column-format distance matrices were calculated by default option (one gap) and were assigned to OTUs using OptiClust algorithm (Westcott and Schloss 2017). The consensus classification for each ASV and OTU clustering units was performed using a naïve Bayesian classifier (Wang et al. 2007) trained using the CIMPhy18 constructed reference database, with a 90% bootstrap confidence threshold. Two ASV and OTU tables were built: one containing all informations, regarding read number present in a cluster (ASVs, 97%-OTUs or 99%-OTUs) and the second, including only clustering units represented with five of more reads per cluster (ASVs*, 97%-OTUs* or 99%-OTUs*). Species scientific names and corresponding authors were checked according to AlgaeBase (www.algaebase.org) prior to checklist formation. All downstream analyses were performed using R Statistical Software (v.4.2.3; R Core Team 2023) and data visualisations were made using ‘ggplot2’ (Wickham 2009) and ‘ggtree’ (Yu et al. 2017) Rpackages and assembled using ‘aplot’ (Yu 2022) Rpackage.

## Checklists

### Metabarcode-based phytoplankton checklist 2020-2021, northern Adriatic.

#### Chlorophyceae

F5B804AA-24C9-502F-ABA8-86FF08623D83

##### Materials

- **Type status:** Other material. **Occurrence:** individualCount: 16; occurrenceID: 9706DA38-BFF2-5D10-BC68-B8A1581D98E0; **Location:** waterBody: Adriatic Sea; country: Croatia; locality: RV001; verbatimDepth: 0-25 m; minimumDepthInMeters: 0; maximumDepthInMeters: 25; locationRemarks: Long term observatory; verbatimLatitude: 45 4 48N; verbatimLongitude: 13d 36' 36'' E; verbatimSRS: WGS84; coordinatePrecision: 0.00001
- **Type status:** Other material. **Occurrence:** individualCount: 15; occurrenceID: 2B743E3C-5049-5B75-867C-95F1607CE2B2; **Location:** waterBody: Adriatic Sea; country: Croatia; locality: RV004; verbatimDepth: 0-25 m; minimumDepthInMeters: 0; maximumDepthInMeters: 25; locationRemarks: Long term observatory; verbatimLatitude: 45 3 42.66N; verbatimLongitude: 13d 32' 56.976'' E; verbatimSRS: WGS84; coordinatePrecision: 0.00001

##### Notes

The sequences listed under this taxon were classified into Chlorophyceae; however, no reference sequence with high enough similarity was available for determining the genus of the respective sequences.

#### Bathycoccus prasinos

W.Eikrem & J.Throndsen, 1990

32195C2E-C349-5F60-9C35-7256CB241BB5

##### Materials

- **Type status:** Other material. **Occurrence:** individualCount: 3; occurrenceID: E5C3E55F-36B2-5998-85EE-D9173CF2FEB0; **Location:** waterBody: Adriatic Sea; country: Croatia; locality: RV001; verbatimDepth: 0-25 m; minimumDepthInMeters: 0; maximumDepthInMeters: 25; locationRemarks: Long term observatory; verbatimLatitude: 45 4 48N; verbatimLongitude: 13d 36' 36'' E; verbatimSRS: WGS84; coordinatePrecision: 0.00001
- **Type status:** Other material. **Occurrence:** individualCount: 2; occurrenceID: CD6B2267-1514-532B-B8D1-676AB51B0321; **Location:** waterBody: Adriatic Sea; country: Croatia; locality: RV004; verbatimDepth: 0-25 m; minimumDepthInMeters: 0; maximumDepthInMeters: 25; locationRemarks: Long term observatory; verbatimLatitude: 45 3 42.66N; verbatimLongitude: 13d 32' 56.976'' E; verbatimSRS: WGS84; coordinatePrecision: 0.00001

#### Tetraselmis marina

(Cienkowski) R.E.Norris, Hori & Chihara, 1980

B456AA88-4AAF-51CB-AB6F-1C850DB7D18E

##### Materials

- **Type status:** Other material. **Occurrence:** individualCount: 242; occurrenceID: CA8A585E-8D31-5C86-93D8-DFD730205702; **Location:** waterBody: Adriatic Sea; country: Croatia; locality: RV001; verbatimDepth: 0-25 m; minimumDepthInMeters: 0; maximumDepthInMeters: 25; locationRemarks: Long term observatory; verbatimLatitude: 45 4 48N; verbatimLongitude: 13d 36' 36'' E; verbatimSRS: WGS84; coordinatePrecision: 0.00001
- **Type status:** Other material. **Occurrence:** individualCount: 1; occurrenceID: 45E39C3E-9077-5268-A6B1-6DDAFD7962A0; **Location:** waterBody: Adriatic Sea; country: Croatia; locality: RV004; verbatimDepth: 0-25 m; minimumDepthInMeters: 0; maximumDepthInMeters: 25; locationRemarks: Long term observatory; verbatimLatitude: 45 3 42.66N; verbatimLongitude: 13d 32' 56.976'' E; verbatimSRS: WGS84; coordinatePrecision: 0.00001

#### Tetraselmis sp.

56DBDE04-F9E0-500F-B4EB-BBF783925B12

##### Materials

- **Type status:** Other material. **Occurrence:** individualCount: 4; occurrenceID: 10D61495-7ECB-54D0-9A1B-4509BE42CBA4; **Location:** waterBody: Adriatic Sea; country: Croatia; locality: RV001; verbatimDepth: 0-25 m; minimumDepthInMeters: 0; maximumDepthInMeters: 25; locationRemarks: Long term observatory; verbatimLatitude: 45 4 48N; verbatimLongitude: 13d 36' 36'' E; verbatimSRS: WGS84; coordinatePrecision: 0.00001
- **Type status:** Other material. **Occurrence:** individualCount: 5; occurrenceID: CB283C6E-70B1-5AD3-AAF5-7786EE6FBB66; **Location:** waterBody: Adriatic Sea; country: Croatia; locality: RV004; verbatimDepth: 0-25 m; minimumDepthInMeters: 0; maximumDepthInMeters: 25; locationRemarks: Long term observatory; verbatimLatitude: 45 3 42.66N; verbatimLongitude: 13d 32' 56.976'' E; verbatimSRS: WGS84; coordinatePrecision: 0.00001

#### Pycnococcus provasolii

R.R.L.Guillard, 1991

B037D58B-5E4C-51E3-8C9D-1FD264F027B7

##### Materials

- **Type status:** Other material. **Occurrence:** individualCount: 5; occurrenceID: 5383EC68-19F1-519C-BF8D-9CDBB6710670; **Location:** waterBody: Adriatic Sea; country: Croatia; locality: RV001; verbatimDepth: 0-25 m; minimumDepthInMeters: 0; maximumDepthInMeters: 25; locationRemarks: Long term observatory; verbatimLatitude: 45 4 48N; verbatimLongitude: 13d 36' 36'' E; verbatimSRS: WGS84; coordinatePrecision: 0.00001
- **Type status:** Other material. **Occurrence:** individualCount: 7; occurrenceID: 28FBBF51-A093-5900-BBCA-17D6D297F6DC; **Location:** waterBody: Adriatic Sea; country: Croatia; locality: RV004; verbatimDepth: 0-25 m; minimumDepthInMeters: 0; maximumDepthInMeters: 25; locationRemarks: Long term observatory; verbatimLatitude: 45 3 42.66N; verbatimLongitude: 13d 32' 56.976'' E; verbatimSRS: WGS84; coordinatePrecision: 0.00001

#### Cryptophyceae

80149AFF-E63A-55B9-AAF5-4E7060956941

##### Materials

- **Type status:** Other material. **Occurrence:** individualCount: 10; occurrenceID: 45F24487-967A-5D53-AD67-75981B57947C; **Location:** waterBody: Adriatic Sea; country: Croatia; locality: RV001; verbatimDepth: 0-25 m; minimumDepthInMeters: 0; maximumDepthInMeters: 25; locationRemarks: Long term observatory; verbatimLatitude: 45 4 48N; verbatimLongitude: 13d 36' 36'' E; verbatimSRS: WGS84; coordinatePrecision: 0.00001

##### Notes

The sequences listed under this taxon were classified into Cryptophyceae; however, no reference sequence with high enough similarity was available for determining the genus of the respective sequences.

#### Cryptomonas sp.

4B6E3BE7-CAB1-580A-B60F-71855E422102

##### Materials

- **Type status:** Other material. **Occurrence:** individualCount: 13; occurrenceID: E815DBB2-9B1A-5B7D-91F0-A592262BA538; **Location:** waterBody: Adriatic Sea; country: Croatia; locality: RV001; verbatimDepth: 0-25 m; minimumDepthInMeters: 0; maximumDepthInMeters: 25; locationRemarks: Long term observatory; verbatimLatitude: 45 4 48N; verbatimLongitude: 13d 36' 36'' E; verbatimSRS: WGS84; coordinatePrecision: 0.00001
- **Type status:** Other material. **Occurrence:** individualCount: 8; occurrenceID: 7B40F1E6-FBB8-5EC8-9DCB-0189760887F2; **Location:** waterBody: Adriatic Sea; country: Croatia; locality: RV004; verbatimDepth: 0-25 m; minimumDepthInMeters: 0; maximumDepthInMeters: 25; locationRemarks: Long term observatory; verbatimLatitude: 45 3 42.66N; verbatimLongitude: 13d 32' 56.976'' E; verbatimSRS: WGS84; coordinatePrecision: 0.00001

#### Teleaulax amphioxeia

(W.Conrad) D.R.A.Hill, 1992

EAEB4BF3-08E9-5FF4-83B2-AC23A24BDA7F

##### Materials

- **Type status:** Other material. **Occurrence:** individualCount: 1; occurrenceID: D7EB8996-BCC2-5D4E-8A55-2CA04AADF847; **Location:** waterBody: Adriatic Sea; country: Croatia; locality: RV004; verbatimDepth: 0-25 m; minimumDepthInMeters: 0; maximumDepthInMeters: 25; locationRemarks: Long term observatory; verbatimLatitude: 45 3 42.66N; verbatimLongitude: 13d 32' 56.976'' E; verbatimSRS: WGS84; coordinatePrecision: 0.00001

#### Teleaulax gracilis

Laza-Martinez, 2012

98618EF0-98F1-50AE-B638-8D103787E7DB

##### Materials

- **Type status:** Other material. **Occurrence:** individualCount: 1; occurrenceID: FC15DE29-EBDD-52EA-9EA8-7DE08697E661; **Location:** waterBody: Adriatic Sea; country: Croatia; locality: RV001; verbatimDepth: 0-25 m; minimumDepthInMeters: 0; maximumDepthInMeters: 25; locationRemarks: Long term observatory; verbatimLatitude: 45 4 48N; verbatimLongitude: 13d 36' 36'' E; verbatimSRS: WGS84; coordinatePrecision: 0.00001

#### Pavlova pinguis

J.C.Green, 1967

0FE2992D-7440-57D1-995F-9FAC855D4A2B

##### Materials

- **Type status:** Other material. **Occurrence:** individualCount: 7; occurrenceID: 34CD6E77-701E-501C-BA75-3128F3C2B7E1; **Location:** waterBody: Adriatic Sea; country: Croatia; locality: RV001; verbatimDepth: 0-25 m; minimumDepthInMeters: 0; maximumDepthInMeters: 25; locationRemarks: Long term observatory; verbatimLatitude: 45 4 48N; verbatimLongitude: 13d 36' 36'' E; verbatimSRS: WGS84; coordinatePrecision: 0.00001

#### Pavlovophyceae

44B83171-D57A-5F8E-9406-348C4391EE1B

##### Materials

- **Type status:** Other material. **Occurrence:** individualCount: 1; occurrenceID: 3AFD4C2B-6D68-5243-9E2C-4A3B2828E28B; **Location:** waterBody: Adriatic Sea; country: Croatia; locality: RV001; verbatimDepth: 0-25 m; minimumDepthInMeters: 0; maximumDepthInMeters: 25; locationRemarks: Long term observatory; verbatimLatitude: 45 4 48N; verbatimLongitude: 13d 36' 36'' E; verbatimSRS: WGS84; coordinatePrecision: 0.00001

##### Notes

The sequences listed under this taxon were classified into Pavlovophyceae; however, no reference sequence with high enough similarity was available for determining the genus of the respective sequences.

#### Gephyrocapsa oceanica

(Stosch) Loeblich III, 1980

8B7BEA48-B86C-583C-A7C7-FB6699A80E55

##### Materials

- **Type status:** Other material. **Occurrence:** individualCount: 1; occurrenceID: B0E11BEE-BE29-544C-A577-C90ACD16C7B7; **Location:** waterBody: Adriatic Sea; country: Croatia; locality: RV001; verbatimDepth: 0-25 m; minimumDepthInMeters: 0; maximumDepthInMeters: 25; locationRemarks: Long term observatory; verbatimLatitude: 45 4 48N; verbatimLongitude: 13d 36' 36'' E; verbatimSRS: WGS84; coordinatePrecision: 0.00001

#### Phaeocystis globosa

Scherffel, 1899

6A6EAA48-FF39-5D3C-A180-90232E26EC71

##### Materials

- **Type status:** Other material. **Occurrence:** individualCount: 1; occurrenceID: 5F1978C5-5E26-5403-A7A8-0B26345494D3; **Location:** waterBody: Adriatic Sea; country: Croatia; locality: RV001; verbatimDepth: 0-25 m; minimumDepthInMeters: 0; maximumDepthInMeters: 25; locationRemarks: Long term observatory; verbatimLatitude: 45 4 48N; verbatimLongitude: 13d 36' 36'' E; verbatimSRS: WGS84; coordinatePrecision: 0.00001

#### Phaeocystis sp.

EAF19639-502D-56D2-B566-87D3AE5E6BF3

##### Materials

- **Type status:** Other material. **Occurrence:** individualCount: 2; occurrenceID: 04BA5AB2-6EA4-5A21-823C-C056E993EAFE; **Location:** waterBody: Adriatic Sea; country: Croatia; locality: RV001; verbatimDepth: 0-25 m; minimumDepthInMeters: 0; maximumDepthInMeters: 25; locationRemarks: Long term observatory; verbatimLatitude: 45 4 48N; verbatimLongitude: 13d 36' 36'' E; verbatimSRS: WGS84; coordinatePrecision: 0.00001
- **Type status:** Other material. **Occurrence:** individualCount: 0; occurrenceID: 5958C2B2-9631-5CBB-B545-9BD4CD470CF5; **Location:** waterBody: Adriatic Sea; country: Croatia; locality: RV004; verbatimDepth: 0-25 m; minimumDepthInMeters: 0; maximumDepthInMeters: 25; locationRemarks: Long term observatory; verbatimLatitude: 45 3 42.66N; verbatimLongitude: 13d 32' 56.976'' E; verbatimSRS: WGS84; coordinatePrecision: 0.00001

#### Prymnesium sp.

2118DBE1-15CC-50FD-9E6B-847033B0212D

##### Materials

- **Type status:** Other material. **Occurrence:** individualCount: 9; occurrenceID: F82935C5-B8CB-556D-8974-581F63BC85F2; **Location:** waterBody: Adriatic Sea; country: Croatia; locality: RV004; verbatimDepth: 0-25 m; minimumDepthInMeters: 0; maximumDepthInMeters: 25; locationRemarks: Long term observatory; verbatimLatitude: 45 3 42.66N; verbatimLongitude: 13d 32' 56.976'' E; verbatimSRS: WGS84; coordinatePrecision: 0.00001

#### Prymnesiales

B68F9B12-8399-585C-98FB-5015D915858A

##### Materials

- **Type status:** Other material. **Occurrence:** individualCount: 1; occurrenceID: F79F8D56-B3DC-5063-9666-DEB671F5AB81; **Location:** waterBody: Adriatic Sea; country: Croatia; locality: RV004; verbatimDepth: 0-25 m; minimumDepthInMeters: 0; maximumDepthInMeters: 25; locationRemarks: Long term observatory; verbatimLatitude: 45 3 42.66N; verbatimLongitude: 13d 32' 56.976'' E; verbatimSRS: WGS84; coordinatePrecision: 0.00001

##### Notes

The sequences listed under this taxon were classified into Prymnesiales; however, no reference sequence with high enough similarity was available for determining the genus of the respective sequences.

#### Dinophyceae

1FFBEB39-9808-51E0-A4A4-6CFE6153B208

##### Materials

- **Type status:** Other material. **Occurrence:** individualCount: 131; occurrenceID: D9EB997B-48A3-5082-9705-E6993BBA9BF1; **Location:** waterBody: Adriatic Sea; country: Croatia; locality: RV001; verbatimDepth: 0-25 m; minimumDepthInMeters: 0; maximumDepthInMeters: 25; locationRemarks: Long term observatory; verbatimLatitude: 45 4 48N; verbatimLongitude: 13d 36' 36'' E; verbatimSRS: WGS84; coordinatePrecision: 0.00001
- **Type status:** Other material. **Occurrence:** individualCount: 82; occurrenceID: 168174CA-FF32-5BE7-94D0-D01BA96FD56D; **Location:** waterBody: Adriatic Sea; country: Croatia; locality: RV004; verbatimDepth: 0-25 m; minimumDepthInMeters: 0; maximumDepthInMeters: 25; locationRemarks: Long term observatory; verbatimLatitude: 45 3 42.66N; verbatimLongitude: 13d 32' 56.976'' E; verbatimSRS: WGS84; coordinatePrecision: 0.00001

##### Notes

The sequences listed under this taxon were classified into Dinophyceae; however, no reference sequence with high enough similarity was available for determining the genus of the respective sequences.

#### Amphidinium massartii

Biecheler, 1952

2941E7FD-5B32-5225-B654-21C4CEA8597B

##### Materials

- **Type status:** Other material. **Occurrence:** individualCount: 1; occurrenceID: 80CED4BA-910A-5F2C-914B-429310D3B379; **Location:** waterBody: Adriatic Sea; country: Croatia; locality: RV004; verbatimDepth: 0-25 m; minimumDepthInMeters: 0; maximumDepthInMeters: 25; locationRemarks: Long term observatory; verbatimLatitude: 45 3 42.66N; verbatimLongitude: 13d 32' 56.976'' E; verbatimSRS: WGS84; coordinatePrecision: 0.00001

#### Amphidinium sp.

D8B7B6C0-E196-53E6-8D68-581284C48AEF

##### Materials

- **Type status:** Other material. **Occurrence:** individualCount: 2; occurrenceID: 272C29BC-EE03-5210-9E48-D6AA996F7837; **Location:** waterBody: Adriatic Sea; country: Croatia; locality: RV004; verbatimDepth: 0-25 m; minimumDepthInMeters: 0; maximumDepthInMeters: 25; locationRemarks: Long term observatory; verbatimLatitude: 45 3 42.66N; verbatimLongitude: 13d 32' 56.976'' E; verbatimSRS: WGS84; coordinatePrecision: 0.00001

#### Blastodinium mangini

Chatton, 1908

153C3AB8-5002-5483-969E-75491865F9F5

##### Materials

- **Type status:** Other material. **Occurrence:** individualCount: 3; occurrenceID: 19C0CD1A-99E6-5307-9C3D-6AD61CC9754E; **Location:** waterBody: Adriatic Sea; country: Croatia; locality: RV001; verbatimDepth: 0-25 m; minimumDepthInMeters: 0; maximumDepthInMeters: 25; locationRemarks: Long term observatory; verbatimLatitude: 45 4 48N; verbatimLongitude: 13d 36' 36'' E; verbatimSRS: WGS84; coordinatePrecision: 0.00001
- **Type status:** Other material. **Occurrence:** individualCount: 25; occurrenceID: 7C18A4A1-6B82-5195-9440-5981F9C7F093; **Location:** waterBody: Adriatic Sea; country: Croatia; locality: RV004; verbatimDepth: 0-25 m; minimumDepthInMeters: 0; maximumDepthInMeters: 25; locationRemarks: Long term observatory; verbatimLatitude: 45 3 42.66N; verbatimLongitude: 13d 32' 56.976'' E; verbatimSRS: WGS84; coordinatePrecision: 0.00001

#### Blastodinium spinulosum

Chatton, 1912

83AD5072-F10D-54AC-B200-320E0342D748

##### Materials

- **Type status:** Other material. **Occurrence:** individualCount: 11; occurrenceID: 872F81E1-9427-5C9C-B14E-48E371E86415; **Location:** waterBody: Adriatic Sea; country: Croatia; locality: RV001; verbatimDepth: 0-25 m; minimumDepthInMeters: 0; maximumDepthInMeters: 25; locationRemarks: Long term observatory; verbatimLatitude: 45 4 48N; verbatimLongitude: 13d 36' 36'' E; verbatimSRS: WGS84; coordinatePrecision: 0.00001
- **Type status:** Other material. **Occurrence:** individualCount: 12; occurrenceID: 01543536-9064-5C16-A3AB-8C3D0A21326E; **Location:** waterBody: Adriatic Sea; country: Croatia; locality: RV004; verbatimDepth: 0-25 m; minimumDepthInMeters: 0; maximumDepthInMeters: 25; locationRemarks: Long term observatory; verbatimLatitude: 45 3 42.66N; verbatimLongitude: 13d 32' 56.976'' E; verbatimSRS: WGS84; coordinatePrecision: 0.00001

#### Oodinium sp.

9A69C191-9F28-5A3F-9EC8-50BE540363A0

##### Materials

- **Type status:** Other material. **Occurrence:** individualCount: 10; occurrenceID: 3B0F0D26-B48F-5107-B029-D9E9ADFA6C75; **Location:** waterBody: Adriatic Sea; country: Croatia; locality: RV001; verbatimDepth: 0-25 m; minimumDepthInMeters: 0; maximumDepthInMeters: 25; locationRemarks: Long term observatory; verbatimLatitude: 45 4 48N; verbatimLongitude: 13d 36' 36'' E; verbatimSRS: WGS84; coordinatePrecision: 0.00001

#### Chytriodinium sp.

CC196985-4E0E-54E4-A854-E2114A10644F

##### Materials

- **Type status:** Other material. **Occurrence:** individualCount: 3; occurrenceID: 06AAD71B-49C9-5193-B812-5E8A4095B23C; **Location:** waterBody: Adriatic Sea; country: Croatia; locality: RV001; verbatimDepth: 0-25 m; minimumDepthInMeters: 0; maximumDepthInMeters: 25; locationRemarks: Long term observatory; verbatimLatitude: 45 4 48N; verbatimLongitude: 13d 36' 36'' E; verbatimSRS: WGS84; coordinatePrecision: 0.00001
- **Type status:** Other material. **Occurrence:** individualCount: 3; occurrenceID: 6FB6E707-1065-5906-82F0-80F53E53BC22; **Location:** waterBody: Adriatic Sea; country: Croatia; locality: RV004; verbatimDepth: 0-25 m; minimumDepthInMeters: 0; maximumDepthInMeters: 25; locationRemarks: Long term observatory; verbatimLatitude: 45 3 42.66N; verbatimLongitude: 13d 32' 56.976'' E; verbatimSRS: WGS84; coordinatePrecision: 0.00001

#### Dinophysis acuminata

Claperède & Lachmann, 1859

8FAC4AD1-B084-503B-BDA4-95C504D567AC

##### Materials

- **Type status:** Other material. **Occurrence:** individualCount: 21; occurrenceID: E3A2FD9D-5409-54FD-800E-700EFA46E2D0; **Location:** waterBody: Adriatic Sea; country: Croatia; locality: RV001; verbatimDepth: 0-25 m; minimumDepthInMeters: 0; maximumDepthInMeters: 25; locationRemarks: Long term observatory; verbatimLatitude: 45 4 48N; verbatimLongitude: 13d 36' 36'' E; verbatimSRS: WGS84; coordinatePrecision: 0.00001
- **Type status:** Other material. **Occurrence:** individualCount: 2; occurrenceID: 77D1F0C8-2A97-5462-88A9-871AA567C71F; **Location:** waterBody: Adriatic Sea; country: Croatia; locality: RV004; verbatimDepth: 0-25 m; minimumDepthInMeters: 0; maximumDepthInMeters: 25; locationRemarks: Long term observatory; verbatimLatitude: 45 3 42.66N; verbatimLongitude: 13d 32' 56.976'' E; verbatimSRS: WGS84; coordinatePrecision: 0.00001

#### Tripos candelabrus

(Ehrenberg) F.Gómez, 2013

5A8AFEC9-12F0-59EC-AA64-20E168B2EF4A

##### Materials

- **Type status:** Other material. **Occurrence:** individualCount: 1; occurrenceID: 0402D2AF-DA10-5506-9B8F-07E5D69C4952; **Location:** waterBody: Adriatic Sea; country: Croatia; locality: RV001; verbatimDepth: 0-25 m; minimumDepthInMeters: 0; maximumDepthInMeters: 25; locationRemarks: Long term observatory; verbatimLatitude: 45 4 48N; verbatimLongitude: 13d 36' 36'' E; verbatimSRS: WGS84; coordinatePrecision: 0.00001

#### Tripos digitatus

(F.Schütt) F.Gómez, 2013

3A9152B0-F3C0-5496-A155-135562A61B15

##### Materials

- **Type status:** Other material. **Occurrence:** individualCount: 12; occurrenceID: 52723F08-E675-5663-8335-75AE3801D5DD; **Location:** waterBody: Adriatic Sea; country: Croatia; locality: RV001; verbatimDepth: 0-25 m; minimumDepthInMeters: 0; maximumDepthInMeters: 25; locationRemarks: Long term observatory; verbatimLatitude: 45 4 48N; verbatimLongitude: 13d 36' 36'' E; verbatimSRS: WGS84; coordinatePrecision: 0.00001

#### Tripos extensus

(Gourret) F.Gómez, 2013

5777BE7B-339A-5FC5-A8E2-C837B205957E

##### Materials

- **Type status:** Other material. **Occurrence:** individualCount: 4; occurrenceID: 707D0F78-F715-5E81-BC98-B8B3D47F3BF2; **Location:** waterBody: Adriatic Sea; country: Croatia; locality: RV001; verbatimDepth: 0-25 m; minimumDepthInMeters: 0; maximumDepthInMeters: 25; locationRemarks: Long term observatory; verbatimLatitude: 45 4 48N; verbatimLongitude: 13d 36' 36'' E; verbatimSRS: WGS84; coordinatePrecision: 0.00001
- **Type status:** Other material. **Occurrence:** individualCount: 5; occurrenceID: 3F5FEC46-9A5C-5540-AA6E-FA60AA755BCF; **Location:** waterBody: Adriatic Sea; country: Croatia; locality: RV004; verbatimDepth: 0-25 m; minimumDepthInMeters: 0; maximumDepthInMeters: 25; locationRemarks: Long term observatory; verbatimLatitude: 45 3 42.66N; verbatimLongitude: 13d 32' 56.976'' E; verbatimSRS: WGS84; coordinatePrecision: 0.00001

#### Tripos hexacanthus

(Gourret) F.Gómez, 2013

D3187E0C-A7ED-5086-ABFB-1CA66222A5FC

##### Materials

- **Type status:** Other material. **Occurrence:** individualCount: 91; occurrenceID: 8429077C-064E-5BAB-AE34-A8EBC2F1C3E6; **Location:** waterBody: Adriatic Sea; country: Croatia; locality: RV001; verbatimDepth: 0-25 m; minimumDepthInMeters: 0; maximumDepthInMeters: 25; locationRemarks: Long term observatory; verbatimLatitude: 45 4 48N; verbatimLongitude: 13d 36' 36'' E; verbatimSRS: WGS84; coordinatePrecision: 0.00001
- **Type status:** Other material. **Occurrence:** individualCount: 40; occurrenceID: B5861388-C9A1-5B1B-8CEB-427C11427B4B; **Location:** waterBody: Adriatic Sea; country: Croatia; locality: RV004; verbatimDepth: 0-25 m; minimumDepthInMeters: 0; maximumDepthInMeters: 25; locationRemarks: Long term observatory; verbatimLatitude: 45 3 42.66N; verbatimLongitude: 13d 32' 56.976'' E; verbatimSRS: WGS84; coordinatePrecision: 0.00001

#### Tripos massiliensis

(Gourret) F.Gómez, 2013

205DCCB4-4336-57E0-B382-7DA5C88E4E0D

##### Materials

- **Type status:** Other material. **Occurrence:** individualCount: 19; occurrenceID: B3B605F2-D3F5-5A37-8F93-5D2F59AD36AD; **Location:** waterBody: Adriatic Sea; country: Croatia; locality: RV001; verbatimDepth: 0-25 m; minimumDepthInMeters: 0; maximumDepthInMeters: 25; locationRemarks: Long term observatory; verbatimLatitude: 45 4 48N; verbatimLongitude: 13d 36' 36'' E; verbatimSRS: WGS84; coordinatePrecision: 0.00001
- **Type status:** Other material. **Occurrence:** individualCount: 4; occurrenceID: 68EB2549-7C2A-5210-8774-E48ADD797AC9; **Location:** waterBody: Adriatic Sea; country: Croatia; locality: RV004; verbatimDepth: 0-25 m; minimumDepthInMeters: 0; maximumDepthInMeters: 25; locationRemarks: Long term observatory; verbatimLatitude: 45 3 42.66N; verbatimLongitude: 13d 32' 56.976'' E; verbatimSRS: WGS84; coordinatePrecision: 0.00001

#### Tripos sp.

D053239A-92DA-5BA7-91E6-C46A4DC94411

##### Materials

- **Type status:** Other material. **Occurrence:** individualCount: 328; occurrenceID: 4CFC660E-ED20-56B0-8F0E-494442095093; **Location:** waterBody: Adriatic Sea; country: Croatia; locality: RV001; verbatimDepth: 0-25 m; minimumDepthInMeters: 0; maximumDepthInMeters: 25; locationRemarks: Long term observatory; verbatimLatitude: 45 4 48N; verbatimLongitude: 13d 36' 36'' E; verbatimSRS: WGS84; coordinatePrecision: 0.00001
- **Type status:** Other material. **Occurrence:** individualCount: 151; occurrenceID: 53ADE75D-B61F-51BE-B68C-FA9E03EBD921; **Location:** waterBody: Adriatic Sea; country: Croatia; locality: RV004; verbatimDepth: 0-25 m; minimumDepthInMeters: 0; maximumDepthInMeters: 25; locationRemarks: Long term observatory; verbatimLatitude: 45 3 42.66N; verbatimLongitude: 13d 32' 56.976'' E; verbatimSRS: WGS84; coordinatePrecision: 0.00001

#### Amylax triacantha

(Jørgensen) Sournia, 1984

4B202B18-6952-5F7E-AB23-53C4739F3B05

##### Materials

- **Type status:** Other material. **Occurrence:** individualCount: 2; occurrenceID: 23A2DCF9-9623-5663-A82A-F8A27F49FCD5; **Location:** waterBody: Adriatic Sea; country: Croatia; locality: RV001; verbatimDepth: 0-25 m; minimumDepthInMeters: 0; maximumDepthInMeters: 25; locationRemarks: Long term observatory; verbatimLatitude: 45 4 48N; verbatimLongitude: 13d 36' 36'' E; verbatimSRS: WGS84; coordinatePrecision: 0.00001

#### Gonyaulax ellegaardiae

Diesing, 1866

EFC12BD4-FB21-592D-967A-A4D0FF13FDAC

##### Materials

- **Type status:** Other material. **Occurrence:** individualCount: 1; occurrenceID: DBECE0C9-9B2D-5E24-8FF7-82465A56CCAC; **Location:** waterBody: Adriatic Sea; country: Croatia; locality: RV001; verbatimDepth: 0-25 m; minimumDepthInMeters: 0; maximumDepthInMeters: 25; locationRemarks: Long term observatory; verbatimLatitude: 45 4 48N; verbatimLongitude: 13d 36' 36'' E; verbatimSRS: WGS84; coordinatePrecision: 0.00001
- **Type status:** Other material. **Occurrence:** individualCount: 0; occurrenceID: 71A44FA2-437A-5CBC-AC88-11FA03C313E6; **Location:** waterBody: Adriatic Sea; country: Croatia; locality: RV004; verbatimDepth: 0-25 m; minimumDepthInMeters: 0; maximumDepthInMeters: 25; locationRemarks: Long term observatory; verbatimLatitude: 45 3 42.66N; verbatimLongitude: 13d 32' 56.976'' E; verbatimSRS: WGS84; coordinatePrecision: 0.00001

#### Gonyaulax sp.

F0D4D604-0A5C-52C0-A0E8-C96BDD829822

##### Materials

- **Type status:** Other material. **Occurrence:** individualCount: 32; occurrenceID: B324628C-89FF-507B-8554-31C310691FBF; **Location:** waterBody: Adriatic Sea; country: Croatia; locality: RV001; verbatimDepth: 0-25 m; minimumDepthInMeters: 0; maximumDepthInMeters: 25; locationRemarks: Long term observatory; verbatimLatitude: 45 4 48N; verbatimLongitude: 13d 36' 36'' E; verbatimSRS: WGS84; coordinatePrecision: 0.00001
- **Type status:** Other material. **Occurrence:** individualCount: 11; occurrenceID: 7B2FE0A6-318E-5071-BE4D-D250C8237C57; **Location:** waterBody: Adriatic Sea; country: Croatia; locality: RV004; verbatimDepth: 0-25 m; minimumDepthInMeters: 0; maximumDepthInMeters: 25; locationRemarks: Long term observatory; verbatimLatitude: 45 3 42.66N; verbatimLongitude: 13d 32' 56.976'' E; verbatimSRS: WGS84; coordinatePrecision: 0.00001

#### Impagidinium pallidum

J.P.Bujak, 1984

1031B1AC-EE3C-5800-B650-B4963B0907A9

##### Materials

- **Type status:** Other material. **Occurrence:** individualCount: 1; occurrenceID: AECBF26B-30EB-5A8F-AC3C-7CC5165228FE; **Location:** waterBody: Adriatic Sea; country: Croatia; locality: RV001; verbatimDepth: 0-25 m; minimumDepthInMeters: 0; maximumDepthInMeters: 25; locationRemarks: Long term observatory; verbatimLatitude: 45 4 48N; verbatimLongitude: 13d 36' 36'' E; verbatimSRS: WGS84; coordinatePrecision: 0.00001

#### Lingulodinium polyedra

(F.Stein) J.D.Dodge, 1989

E76EAA9D-B386-5916-9E25-667664693FC3

##### Materials

- **Type status:** Other material. **Occurrence:** individualCount: 2; occurrenceID: C0FDB159-1CA4-59C5-A164-974AA783D137; **Location:** waterBody: Adriatic Sea; country: Croatia; locality: RV001; verbatimDepth: 0-25 m; minimumDepthInMeters: 0; maximumDepthInMeters: 25; locationRemarks: Long term observatory; verbatimLatitude: 45 4 48N; verbatimLongitude: 13d 36' 36'' E; verbatimSRS: WGS84; coordinatePrecision: 0.00001
- **Type status:** Other material. **Occurrence:** individualCount: 3; occurrenceID: 134E7672-2772-5D06-976E-7208DFA66337; **Location:** waterBody: Adriatic Sea; country: Croatia; locality: RV004; verbatimDepth: 0-25 m; minimumDepthInMeters: 0; maximumDepthInMeters: 25; locationRemarks: Long term observatory; verbatimLatitude: 45 3 42.66N; verbatimLongitude: 13d 32' 56.976'' E; verbatimSRS: WGS84; coordinatePrecision: 0.00001

#### Protoceratium reticulatum

(Claparède & Lachmann) Bütschli, 1885

DC8A292C-03FB-5740-BEAB-7720797F51E8

##### Materials

- **Type status:** Other material. **Occurrence:** individualCount: 1; occurrenceID: 6C02356A-963B-59AB-97BC-3F05A8592472; **Location:** waterBody: Adriatic Sea; country: Croatia; locality: RV001; verbatimDepth: 0-25 m; minimumDepthInMeters: 0; maximumDepthInMeters: 25; locationRemarks: Long term observatory; verbatimLatitude: 45 4 48N; verbatimLongitude: 13d 36' 36'' E; verbatimSRS: WGS84; coordinatePrecision: 0.00001
- **Type status:** Other material. **Occurrence:** individualCount: 10; occurrenceID: FF5D63AC-508C-58ED-BA2F-13B5F62A077F; **Location:** waterBody: Adriatic Sea; country: Croatia; locality: RV004; verbatimDepth: 0-25 m; minimumDepthInMeters: 0; maximumDepthInMeters: 25; locationRemarks: Long term observatory; verbatimLatitude: 45 3 42.66N; verbatimLongitude: 13d 32' 56.976'' E; verbatimSRS: WGS84; coordinatePrecision: 0.00001

#### Cucumeridinium coeruleum

(Dogiel) F.Gomez, P.López-García, H.Takayama & D.Moreira, 2015

20BC5D8A-0F35-5DC2-B4F0-D1B9F0994783

##### Materials

- **Type status:** Other material. **Occurrence:** individualCount: 1; occurrenceID: F789548C-6532-5C65-B563-6F67B2E8564F; **Location:** waterBody: Adriatic Sea; country: Croatia; locality: RV001; verbatimDepth: 0-25 m; minimumDepthInMeters: 0; maximumDepthInMeters: 25; locationRemarks: Long term observatory; verbatimLatitude: 45 4 48N; verbatimLongitude: 13d 36' 36'' E; verbatimSRS: WGS84; coordinatePrecision: 0.00001

#### Alexandrium affine

(H.Inoue & Y.Fukuyo) Balech, 1995

CFD83BCE-55D0-5618-BE24-9862CD715DBC

##### Materials

- **Type status:** Other material. **Occurrence:** individualCount: 6; occurrenceID: BE276D76-970B-5673-9BB6-8A40A0FE5C68; **Location:** waterBody: Adriatic Sea; country: Croatia; locality: RV001; verbatimDepth: 0-25 m; minimumDepthInMeters: 0; maximumDepthInMeters: 25; locationRemarks: Long term observatory; verbatimLatitude: 45 4 48N; verbatimLongitude: 13d 36' 36'' E; verbatimSRS: WGS84; coordinatePrecision: 0.00001
- **Type status:** Other material. **Occurrence:** individualCount: 5; occurrenceID: 97B121EE-0710-5F38-9569-1708002D811C; **Location:** waterBody: Adriatic Sea; country: Croatia; locality: RV004; verbatimDepth: 0-25 m; minimumDepthInMeters: 0; maximumDepthInMeters: 25; locationRemarks: Long term observatory; verbatimLatitude: 45 3 42.66N; verbatimLongitude: 13d 32' 56.976'' E; verbatimSRS: WGS84; coordinatePrecision: 0.00001

#### Alexandrium margalefii

Balech, 1994

B911D7DC-FE30-530E-9278-777B25C115AC

##### Materials

- **Type status:** Other material. **Occurrence:** individualCount: 5; occurrenceID: 098B9954-2719-5C29-906B-095073720E41; **Location:** waterBody: Adriatic Sea; country: Croatia; locality: RV001; verbatimDepth: 0-25 m; minimumDepthInMeters: 0; maximumDepthInMeters: 25; locationRemarks: Long term observatory; verbatimLatitude: 45 4 48N; verbatimLongitude: 13d 36' 36'' E; verbatimSRS: WGS84; coordinatePrecision: 0.00001
- **Type status:** Other material. **Occurrence:** individualCount: 27; occurrenceID: C9BD5F6E-1724-51A7-9F44-C4B84EC7917A; **Location:** waterBody: Adriatic Sea; country: Croatia; locality: RV004; verbatimDepth: 0-25 m; minimumDepthInMeters: 0; maximumDepthInMeters: 25; locationRemarks: Long term observatory; verbatimLatitude: 45 3 42.66N; verbatimLongitude: 13d 32' 56.976'' E; verbatimSRS: WGS84; coordinatePrecision: 0.00001

#### Alexandrium tamarense

(Lebour) Balech, 1995

BCB6779B-FD79-528D-ABDE-8C85C1C124A5

##### Materials

- **Type status:** Other material. **Occurrence:** individualCount: 1; occurrenceID: AF899D85-3A87-57DB-861D-FC53E191577D; **Location:** waterBody: Adriatic Sea; country: Croatia; locality: RV004; verbatimDepth: 0-25 m; minimumDepthInMeters: 0; maximumDepthInMeters: 25; locationRemarks: Long term observatory; verbatimLatitude: 45 3 42.66N; verbatimLongitude: 13d 32' 56.976'' E; verbatimSRS: WGS84; coordinatePrecision: 0.00001

#### Fragilidium mexicanum

D.W.Coats, T.R.Bachvaroff & C.F.Delwiche

00242CEE-2DDF-5037-A9C8-8711D2922CD3

##### Materials

- **Type status:** Other material. **Occurrence:** individualCount: 23; occurrenceID: B7126629-6335-57E0-AC32-DCB2984530A8; **Location:** waterBody: Adriatic Sea; country: Croatia; locality: RV001; verbatimDepth: 0-25 m; minimumDepthInMeters: 0; maximumDepthInMeters: 25; locationRemarks: Long term observatory; verbatimLatitude: 45 4 48N; verbatimLongitude: 13d 36' 36'' E; verbatimSRS: WGS84; coordinatePrecision: 0.00001
- **Type status:** Other material. **Occurrence:** individualCount: 212; occurrenceID: 32456D18-A18D-586F-9270-2704CA536538; **Location:** waterBody: Adriatic Sea; country: Croatia; locality: RV004; verbatimDepth: 0-25 m; minimumDepthInMeters: 0; maximumDepthInMeters: 25; locationRemarks: Long term observatory; verbatimLatitude: 45 3 42.66N; verbatimLongitude: 13d 32' 56.976'' E; verbatimSRS: WGS84; coordinatePrecision: 0.00001

#### Fragilidium subglobosum

Balech, 1988

18DADE17-6D11-51C3-B94B-07601E7B0D06

##### Materials

- **Type status:** Other material. **Occurrence:** individualCount: 2; occurrenceID: F1E7455D-ACD9-54D7-86D5-2985293E781B; **Location:** waterBody: Adriatic Sea; country: Croatia; locality: RV001; verbatimDepth: 0-25 m; minimumDepthInMeters: 0; maximumDepthInMeters: 25; locationRemarks: Long term observatory; verbatimLatitude: 45 4 48N; verbatimLongitude: 13d 36' 36'' E; verbatimSRS: WGS84; coordinatePrecision: 0.00001
- **Type status:** Other material. **Occurrence:** individualCount: 0; occurrenceID: AD0267BA-4039-5B8F-B375-A088D63FDF93; **Location:** waterBody: Adriatic Sea; country: Croatia; locality: RV004; verbatimDepth: 0-25 m; minimumDepthInMeters: 0; maximumDepthInMeters: 25; locationRemarks: Long term observatory; verbatimLatitude: 45 3 42.66N; verbatimLongitude: 13d 32' 56.976'' E; verbatimSRS: WGS84; coordinatePrecision: 0.00001

#### Gonyaulacales

0332BB38-71A5-5E0D-86A4-7FC97E183556

##### Materials

- **Type status:** Other material. **Occurrence:** individualCount: 42; occurrenceID: 84355E48-53D2-5118-B609-6B05FF94462B; **Location:** waterBody: Adriatic Sea; country: Croatia; locality: RV001; verbatimDepth: 0-25 m; minimumDepthInMeters: 0; maximumDepthInMeters: 25; locationRemarks: Long term observatory; verbatimLatitude: 45 4 48N; verbatimLongitude: 13d 36' 36'' E; verbatimSRS: WGS84; coordinatePrecision: 0.00001
- **Type status:** Other material. **Occurrence:** individualCount: 20; occurrenceID: 519B74A1-B420-560D-B5F5-357B1C53C299; **Location:** waterBody: Adriatic Sea; country: Croatia; locality: RV004; verbatimDepth: 0-25 m; minimumDepthInMeters: 0; maximumDepthInMeters: 25; locationRemarks: Long term observatory; verbatimLatitude: 45 3 42.66N; verbatimLongitude: 13d 32' 56.976'' E; verbatimSRS: WGS84; coordinatePrecision: 0.00001

##### Notes

The sequences listed under this taxon were classified into Gonyaulacales; however, no reference sequence with high enough similarity was available for determining the genus of the respective sequences.

#### Gymnoxanthella radiolariae

T.Yuasa & T.Horiguchi, 2016

C83F149D-6F2E-5974-A1FB-EB912F7CA38D

##### Materials

- **Type status:** Other material. **Occurrence:** individualCount: 1; occurrenceID: 7B40ABB5-5497-518C-88D7-5758F452210C; **Location:** waterBody: Adriatic Sea; country: Croatia; locality: RV004; verbatimDepth: 0-25 m; minimumDepthInMeters: 0; maximumDepthInMeters: 25; locationRemarks: Long term observatory; verbatimLatitude: 45 3 42.66N; verbatimLongitude: 13d 32' 56.976'' E; verbatimSRS: WGS84; coordinatePrecision: 0.00001

#### Gyrodinium sp.

1F20169B-8E5E-5045-BCBB-417EB202CC25

##### Materials

- **Type status:** Other material. **Occurrence:** individualCount: 13; occurrenceID: 6F9FBF15-B4AA-5573-982E-99B16D6AFFBB; **Location:** waterBody: Adriatic Sea; country: Croatia; locality: RV001; verbatimDepth: 0-25 m; minimumDepthInMeters: 0; maximumDepthInMeters: 25; locationRemarks: Long term observatory; verbatimLatitude: 45 4 48N; verbatimLongitude: 13d 36' 36'' E; verbatimSRS: WGS84; coordinatePrecision: 0.00001
- **Type status:** Other material. **Occurrence:** individualCount: 2; occurrenceID: 025316F3-341C-53DA-8291-912F5D47FD6F; **Location:** waterBody: Adriatic Sea; country: Croatia; locality: RV004; verbatimDepth: 0-25 m; minimumDepthInMeters: 0; maximumDepthInMeters: 25; locationRemarks: Long term observatory; verbatimLatitude: 45 3 42.66N; verbatimLongitude: 13d 32' 56.976'' E; verbatimSRS: WGS84; coordinatePrecision: 0.00001

#### Lepidodinium chlorophorum

(M.Elbrächter & E.Schnepf) Gert Hansen, Botes & Salas, 2007

23AB3EA8-1335-5DBB-8F37-C7B31B8177F6

##### Materials

- **Type status:** Other material. **Occurrence:** individualCount: 3; occurrenceID: 39546B18-C5B4-58DE-BAC8-4D0B2474907C; **Location:** waterBody: Adriatic Sea; country: Croatia; locality: RV001; verbatimDepth: 0-25 m; minimumDepthInMeters: 0; maximumDepthInMeters: 25; locationRemarks: Long term observatory; verbatimLatitude: 45 4 48N; verbatimLongitude: 13d 36' 36'' E; verbatimSRS: WGS84; coordinatePrecision: 0.00001
- **Type status:** Other material. **Occurrence:** individualCount: 6; occurrenceID: F2E870D2-676B-50B3-BC74-F30F5F925A2F; **Location:** waterBody: Adriatic Sea; country: Croatia; locality: RV004; verbatimDepth: 0-25 m; minimumDepthInMeters: 0; maximumDepthInMeters: 25; locationRemarks: Long term observatory; verbatimLatitude: 45 3 42.66N; verbatimLongitude: 13d 32' 56.976'' E; verbatimSRS: WGS84; coordinatePrecision: 0.00001

#### Pelagodinium bei

(H.J.Spero) Siano, Montresor, Probert & Vargas, 2010

5FABEE14-5032-5611-9716-650440E592E7

##### Materials

- **Type status:** Other material. **Occurrence:** individualCount: 5; occurrenceID: 85283173-9511-56F8-B7DB-009CA2AF988D; **Location:** waterBody: Adriatic Sea; country: Croatia; locality: RV004; verbatimDepth: 0-25 m; minimumDepthInMeters: 0; maximumDepthInMeters: 25; locationRemarks: Long term observatory; verbatimLatitude: 45 3 42.66N; verbatimLongitude: 13d 32' 56.976'' E; verbatimSRS: WGS84; coordinatePrecision: 0.00001

#### Karlodinium veneficum

(D.Ballantine) J.Larsen, 2000

90A6DF37-B948-5514-A3EF-30EC42AA2CBF

##### Materials

- **Type status:** Other material. **Occurrence:** individualCount: 1216; occurrenceID: 2038A53A-37F6-54F0-925D-26EED20EA5C2; **Location:** waterBody: Adriatic Sea; country: Croatia; locality: RV001; verbatimDepth: 0-25 m; minimumDepthInMeters: 0; maximumDepthInMeters: 25; locationRemarks: Long term observatory; verbatimLatitude: 45 4 48N; verbatimLongitude: 13d 36' 36'' E; verbatimSRS: WGS84; coordinatePrecision: 0.00001
- **Type status:** Other material. **Occurrence:** individualCount: 402; occurrenceID: E02156A0-F472-5057-9449-54DE855207A9; **Location:** waterBody: Adriatic Sea; country: Croatia; locality: RV004; verbatimDepth: 0-25 m; minimumDepthInMeters: 0; maximumDepthInMeters: 25; locationRemarks: Long term observatory; verbatimLatitude: 45 3 42.66N; verbatimLongitude: 13d 32' 56.976'' E; verbatimSRS: WGS84; coordinatePrecision: 0.00001

#### Balechina pachydermata

(Kofoid & Swezy) A.R.Loeblich Jr. & A.R.Loeblich III, 1968

D3FBB495-F68B-532B-B1DA-908EA4DDE29B

##### Materials

- **Type status:** Other material. **Occurrence:** individualCount: 3; occurrenceID: 844FF6F6-2263-579F-A63B-60DDBB278F04; **Location:** waterBody: Adriatic Sea; country: Croatia; locality: RV004; verbatimDepth: 0-25 m; minimumDepthInMeters: 0; maximumDepthInMeters: 25; locationRemarks: Long term observatory; verbatimLatitude: 45 3 42.66N; verbatimLongitude: 13d 32' 56.976'' E; verbatimSRS: WGS84; coordinatePrecision: 0.00001

#### Gymnodiniales

2E8B1CCB-5D09-5842-AC47-16E2C6B161A3

##### Materials

- **Type status:** Other material. **Occurrence:** individualCount: 7; occurrenceID: 93AC4803-FBFC-57BF-AC2F-DA89CE438889; **Location:** waterBody: Adriatic Sea; country: Croatia; locality: RV001; verbatimDepth: 0-25 m; minimumDepthInMeters: 0; maximumDepthInMeters: 25; locationRemarks: Long term observatory; verbatimLatitude: 45 4 48N; verbatimLongitude: 13d 36' 36'' E; verbatimSRS: WGS84; coordinatePrecision: 0.00001
- **Type status:** Other material. **Occurrence:** individualCount: 2; occurrenceID: 4A0B8982-5FFC-5B52-8061-7DE3A3D63033; **Location:** waterBody: Adriatic Sea; country: Croatia; locality: RV004; verbatimDepth: 0-25 m; minimumDepthInMeters: 0; maximumDepthInMeters: 25; locationRemarks: Long term observatory; verbatimLatitude: 45 3 42.66N; verbatimLongitude: 13d 32' 56.976'' E; verbatimSRS: WGS84; coordinatePrecision: 0.00001

##### Notes

The sequences listed under this taxon were classified into Gymnodiniales; however, no reference sequence with high enough similarity was available for determining the genus of the respective sequences.

#### Kofoidinium cf. pavillardii

J.Cachon & M.Cachon, 1967

39C5225C-9214-5103-AF1F-BD60773488B2

##### Materials

- **Type status:** Other material. **Occurrence:** individualCount: 547; occurrenceID: 662D5308-AB64-527F-BA92-105154589A9B; **Location:** waterBody: Adriatic Sea; country: Croatia; locality: RV001; verbatimDepth: 0-25 m; minimumDepthInMeters: 0; maximumDepthInMeters: 25; locationRemarks: Long term observatory; verbatimLatitude: 45 4 48N; verbatimLongitude: 13d 36' 36'' E; verbatimSRS: WGS84; coordinatePrecision: 0.00001
- **Type status:** Other material. **Occurrence:** individualCount: 140; occurrenceID: 3D984AB3-B125-5733-A2BE-CA9FA1C36387; **Location:** waterBody: Adriatic Sea; country: Croatia; locality: RV004; verbatimDepth: 0-25 m; minimumDepthInMeters: 0; maximumDepthInMeters: 25; locationRemarks: Long term observatory; verbatimLatitude: 45 3 42.66N; verbatimLongitude: 13d 32' 56.976'' E; verbatimSRS: WGS84; coordinatePrecision: 0.00001

#### Noctiluca scintillans

(Macartney) Kofoid & Swezy, 1921

791D9B84-1471-5D2F-A381-EEAFEACD6EB8

##### Materials

- **Type status:** Other material. **Occurrence:** individualCount: 256; occurrenceID: 31BF341D-BB80-523B-B5AB-20B13C7A0602; **Location:** waterBody: Adriatic Sea; country: Croatia; locality: RV001; verbatimDepth: 0-25 m; minimumDepthInMeters: 0; maximumDepthInMeters: 25; locationRemarks: Long term observatory; verbatimLatitude: 45 4 48N; verbatimLongitude: 13d 36' 36'' E; verbatimSRS: WGS84; coordinatePrecision: 0.00001
- **Type status:** Other material. **Occurrence:** individualCount: 7637; occurrenceID: CFD4074B-513E-5F49-A45D-164E30826412; **Location:** waterBody: Adriatic Sea; country: Croatia; locality: RV004; verbatimDepth: 0-25 m; minimumDepthInMeters: 0; maximumDepthInMeters: 25; locationRemarks: Long term observatory; verbatimLatitude: 45 3 42.66N; verbatimLongitude: 13d 32' 56.976'' E; verbatimSRS: WGS84; coordinatePrecision: 0.00001

#### Noctilucales

0F0A8A94-8E2D-594E-B35B-B3250DF634A6

##### Materials

- **Type status:** Other material. **Occurrence:** individualCount: 20; occurrenceID: 54FBA744-1461-5FAA-B3A4-0C5F1D1E0BB8; **Location:** waterBody: Adriatic Sea; country: Croatia; locality: RV001; verbatimDepth: 0-25 m; minimumDepthInMeters: 0; maximumDepthInMeters: 25; locationRemarks: Long term observatory; verbatimLatitude: 45 4 48N; verbatimLongitude: 13d 36' 36'' E; verbatimSRS: WGS84; coordinatePrecision: 0.00001
- **Type status:** Other material. **Occurrence:** individualCount: 191; occurrenceID: D25B378A-B502-5969-BD90-B8FFFB56A3B2; **Location:** waterBody: Adriatic Sea; country: Croatia; locality: RV004; verbatimDepth: 0-25 m; minimumDepthInMeters: 0; maximumDepthInMeters: 25; locationRemarks: Long term observatory; verbatimLatitude: 45 3 42.66N; verbatimLongitude: 13d 32' 56.976'' E; verbatimSRS: WGS84; coordinatePrecision: 0.00001

##### Notes

The sequences listed under this taxon were classified into Noctilucales; however, no reference sequence with high enough similarity was available for determining the genus of the respective sequences.

#### Brandtodinium nutricula

(K.Brandt) Probert & Siano, 2014

D1F74028-7AD2-5B0F-8A39-1FE4C268957A

##### Materials

- **Type status:** Other material. **Occurrence:** individualCount: 2; occurrenceID: B2D73CD6-A6F6-52C2-A8F8-358E32A76799; **Location:** waterBody: Adriatic Sea; country: Croatia; locality: RV001; verbatimDepth: 0-25 m; minimumDepthInMeters: 0; maximumDepthInMeters: 25; locationRemarks: Long term observatory; verbatimLatitude: 45 4 48N; verbatimLongitude: 13d 36' 36'' E; verbatimSRS: WGS84; coordinatePrecision: 0.00001

#### Bysmatrum arenicola

T.Horiguchi & R.N.Pienaar, 2000

CF5CF271-6027-549F-85F3-8EDEBF1D42DA

##### Materials

- **Type status:** Other material. **Occurrence:** individualCount: 140; occurrenceID: CE7E3D78-4DBC-557E-A478-CEF6FB996E76; **Location:** waterBody: Adriatic Sea; country: Croatia; locality: RV001; verbatimDepth: 0-25 m; minimumDepthInMeters: 0; maximumDepthInMeters: 25; locationRemarks: Long term observatory; verbatimLatitude: 45 4 48N; verbatimLongitude: 13d 36' 36'' E; verbatimSRS: WGS84; coordinatePrecision: 0.00001
- **Type status:** Other material. **Occurrence:** individualCount: 28; occurrenceID: EB1BAB38-9201-51F4-B606-6549FD4A15C7; **Location:** waterBody: Adriatic Sea; country: Croatia; locality: RV004; verbatimDepth: 0-25 m; minimumDepthInMeters: 0; maximumDepthInMeters: 25; locationRemarks: Long term observatory; verbatimLatitude: 45 3 42.66N; verbatimLongitude: 13d 32' 56.976'' E; verbatimSRS: WGS84; coordinatePrecision: 0.00001

#### Scrippsiella trochoidea

(F.Stein) A.R.Loeblich III, 1976

4A2A14D1-C420-5203-8833-28066EE6DFB2

##### Materials

- **Type status:** Other material. **Occurrence:** individualCount: 5; occurrenceID: A6874464-C9EE-5E15-B72B-0F161A3189B2; **Location:** waterBody: Adriatic Sea; country: Croatia; locality: RV004; verbatimDepth: 0-25 m; minimumDepthInMeters: 0; maximumDepthInMeters: 25; locationRemarks: Long term observatory; verbatimLatitude: 45 3 42.66N; verbatimLongitude: 13d 32' 56.976'' E; verbatimSRS: WGS84; coordinatePrecision: 0.00001

#### Gotoius excentricus

Mertens, Aydin, Takano, Yamaguchi & Matsuoka, 2015

BF6602C2-5F5B-5AAE-9881-076875B50C12

##### Materials

- **Type status:** Other material. **Occurrence:** individualCount: 2; occurrenceID: 199C64BB-5A34-52DF-B8C5-DFB6998024D8; **Location:** waterBody: Adriatic Sea; country: Croatia; locality: RV001; verbatimDepth: 0-25 m; minimumDepthInMeters: 0; maximumDepthInMeters: 25; locationRemarks: Long term observatory; verbatimLatitude: 45 4 48N; verbatimLongitude: 13d 36' 36'' E; verbatimSRS: WGS84; coordinatePrecision: 0.00001

#### Protoperidinium bipes

(Paulsen) Balech, 1974

46DD7C4D-0F34-57A7-BB83-576AD7ADD482

##### Materials

- **Type status:** Other material. **Occurrence:** individualCount: 1; occurrenceID: 076444EA-9B95-58A9-8627-68914820B269; **Location:** waterBody: Adriatic Sea; country: Croatia; locality: RV004; verbatimDepth: 0-25 m; minimumDepthInMeters: 0; maximumDepthInMeters: 25; locationRemarks: Long term observatory; verbatimLatitude: 45 3 42.66N; verbatimLongitude: 13d 32' 56.976'' E; verbatimSRS: WGS84; coordinatePrecision: 0.00001

#### Protoperidinium depressum

(Bailey) Balech, 1974

CE788439-BA06-599D-AABE-F6F5703FE339

##### Materials

- **Type status:** Other material. **Occurrence:** individualCount: 22; occurrenceID: 528E04A5-2DF6-5D4B-A54C-19F09A9D983D; **Location:** waterBody: Adriatic Sea; country: Croatia; locality: RV001; verbatimDepth: 0-25 m; minimumDepthInMeters: 0; maximumDepthInMeters: 25; locationRemarks: Long term observatory; verbatimLatitude: 45 4 48N; verbatimLongitude: 13d 36' 36'' E; verbatimSRS: WGS84; coordinatePrecision: 0.00001
- **Type status:** Other material. **Occurrence:** individualCount: 16; occurrenceID: 17E7CD56-1B97-59A0-B6AD-A5466614D9D5; **Location:** waterBody: Adriatic Sea; country: Croatia; locality: RV004; verbatimDepth: 0-25 m; minimumDepthInMeters: 0; maximumDepthInMeters: 25; locationRemarks: Long term observatory; verbatimLatitude: 45 3 42.66N; verbatimLongitude: 13d 32' 56.976'' E; verbatimSRS: WGS84; coordinatePrecision: 0.00001

#### Protoperidinium divergens

(Ehrenberg) Balech, 1974

221D6C04-9DCC-5A16-B73B-DE75E943D089

##### Materials

- **Type status:** Other material. **Occurrence:** individualCount: 1; occurrenceID: 83923A70-AB2E-5D7A-8DB3-93D2BD1CA7B0; **Location:** waterBody: Adriatic Sea; country: Croatia; locality: RV001; verbatimDepth: 0-25 m; minimumDepthInMeters: 0; maximumDepthInMeters: 25; locationRemarks: Long term observatory; verbatimLatitude: 45 4 48N; verbatimLongitude: 13d 36' 36'' E; verbatimSRS: WGS84; coordinatePrecision: 0.00001

#### Protoperidinium elegans

(Cleve) Balech, 1974

27C4B1B1-99F0-5A71-AD9D-57DF780A9001

##### Materials

- **Type status:** Other material. **Occurrence:** individualCount: 7; occurrenceID: 12A8112C-122A-57C5-A19A-90D757DAF38A; **Location:** waterBody: Adriatic Sea; country: Croatia; locality: RV001; verbatimDepth: 0-25 m; minimumDepthInMeters: 0; maximumDepthInMeters: 25; locationRemarks: Long term observatory; verbatimLatitude: 45 4 48N; verbatimLongitude: 13d 36' 36'' E; verbatimSRS: WGS84; coordinatePrecision: 0.00001
- **Type status:** Other material. **Occurrence:** individualCount: 1; occurrenceID: 955706E7-9B6A-51FA-AFC0-72C125A0E0C3; **Location:** waterBody: Adriatic Sea; country: Croatia; locality: RV004; verbatimDepth: 0-25 m; minimumDepthInMeters: 0; maximumDepthInMeters: 25; locationRemarks: Long term observatory; verbatimLatitude: 45 3 42.66N; verbatimLongitude: 13d 32' 56.976'' E; verbatimSRS: WGS84; coordinatePrecision: 0.00001

#### Protoperidinium punctulatum

(Paulsen) Balech, 1974

9037BC3D-D736-563F-9A04-6F0E4E4A0AEB

##### Materials

- **Type status:** Other material. **Occurrence:** individualCount: 1; occurrenceID: DC39406D-5977-535E-A6E2-608FD85601C9; **Location:** waterBody: Adriatic Sea; country: Croatia; locality: RV001; verbatimDepth: 0-25 m; minimumDepthInMeters: 0; maximumDepthInMeters: 25; locationRemarks: Long term observatory; verbatimLatitude: 45 4 48N; verbatimLongitude: 13d 36' 36'' E; verbatimSRS: WGS84; coordinatePrecision: 0.00001

#### Protoperidinium sp.

CB245FFE-52CC-5A61-A000-A2481F7B3D63

##### Materials

- **Type status:** Other material. **Occurrence:** individualCount: 36; occurrenceID: CF67AFCE-5169-5773-9E46-D5D2B38164E9; **Location:** waterBody: Adriatic Sea; country: Croatia; locality: RV001; verbatimDepth: 0-25 m; minimumDepthInMeters: 0; maximumDepthInMeters: 25; locationRemarks: Long term observatory; verbatimLatitude: 45 4 48N; verbatimLongitude: 13d 36' 36'' E; verbatimSRS: WGS84; coordinatePrecision: 0.00001
- **Type status:** Other material. **Occurrence:** individualCount: 65; occurrenceID: 06B18686-4562-5C00-B21A-EA56D084B651; **Location:** waterBody: Adriatic Sea; country: Croatia; locality: RV004; verbatimDepth: 0-25 m; minimumDepthInMeters: 0; maximumDepthInMeters: 25; locationRemarks: Long term observatory; verbatimLatitude: 45 3 42.66N; verbatimLongitude: 13d 32' 56.976'' E; verbatimSRS: WGS84; coordinatePrecision: 0.00001

#### Prorocentrum cordatum

(Pavillard) J.Schiller, 1933

2C5115F9-34A2-5B8E-B9E7-0B28535DB3BC

##### Materials

- **Type status:** Other material. **Occurrence:** individualCount: 8; occurrenceID: DEA7DF33-D87F-5A2D-B4CE-9B319FEBC33E; **Location:** waterBody: Adriatic Sea; country: Croatia; locality: RV004; verbatimDepth: 0-25 m; minimumDepthInMeters: 0; maximumDepthInMeters: 25; locationRemarks: Long term observatory; verbatimLatitude: 45 3 42.66N; verbatimLongitude: 13d 32' 56.976'' E; verbatimSRS: WGS84; coordinatePrecision: 0.00001

#### Biecheleria cincta

(Siano, Montresor & Zingone) Siano, 2012

DE344C19-CD29-52EA-899D-C5300E23F945

##### Materials

- **Type status:** Other material. **Occurrence:** individualCount: 1; occurrenceID: 5679A4B2-5CAC-565E-BBE8-ED9EAE725021; **Location:** waterBody: Adriatic Sea; country: Croatia; locality: RV001; verbatimDepth: 0-25 m; minimumDepthInMeters: 0; maximumDepthInMeters: 25; locationRemarks: Long term observatory; verbatimLatitude: 45 4 48N; verbatimLongitude: 13d 36' 36'' E; verbatimSRS: WGS84; coordinatePrecision: 0.00001
- **Type status:** Other material. **Occurrence:** individualCount: 1; occurrenceID: D223251B-02BF-5E71-84EB-71CAE0762F23; **Location:** waterBody: Adriatic Sea; country: Croatia; locality: RV004; verbatimDepth: 0-25 m; minimumDepthInMeters: 0; maximumDepthInMeters: 25; locationRemarks: Long term observatory; verbatimLatitude: 45 3 42.66N; verbatimLongitude: 13d 32' 56.976'' E; verbatimSRS: WGS84; coordinatePrecision: 0.00001

#### Symbiodinium sp.

580828D5-8F28-5ABB-AFC0-C81C6834A6A1

##### Materials

- **Type status:** Other material. **Occurrence:** individualCount: 48; occurrenceID: D337EBF3-D81B-5A9B-9199-621F231443EC; **Location:** waterBody: Adriatic Sea; country: Croatia; locality: RV001; verbatimDepth: 0-25 m; minimumDepthInMeters: 0; maximumDepthInMeters: 25; locationRemarks: Long term observatory; verbatimLatitude: 45 4 48N; verbatimLongitude: 13d 36' 36'' E; verbatimSRS: WGS84; coordinatePrecision: 0.00001
- **Type status:** Other material. **Occurrence:** individualCount: 76; occurrenceID: 66E38510-CE86-5CBC-BAD2-B8E27C56640C; **Location:** waterBody: Adriatic Sea; country: Croatia; locality: RV004; verbatimDepth: 0-25 m; minimumDepthInMeters: 0; maximumDepthInMeters: 25; locationRemarks: Long term observatory; verbatimLatitude: 45 3 42.66N; verbatimLongitude: 13d 32' 56.976'' E; verbatimSRS: WGS84; coordinatePrecision: 0.00001

#### Amoebophrya sp.

A25B1DFB-C9D0-5069-BABD-CB578D2726F2

##### Materials

- **Type status:** Other material. **Occurrence:** individualCount: 3; occurrenceID: E546AAE3-1523-5E41-8E0A-9760232BBA76; **Location:** waterBody: Adriatic Sea; country: Croatia; locality: RV001; verbatimDepth: 0-25 m; minimumDepthInMeters: 0; maximumDepthInMeters: 25; locationRemarks: Long term observatory; verbatimLatitude: 45 4 48N; verbatimLongitude: 13d 36' 36'' E; verbatimSRS: WGS84; coordinatePrecision: 0.00001
- **Type status:** Other material. **Occurrence:** individualCount: 32; occurrenceID: 72C92E5A-7C1B-5EAB-8798-9B62B1D2FB9B; **Location:** waterBody: Adriatic Sea; country: Croatia; locality: RV004; verbatimDepth: 0-25 m; minimumDepthInMeters: 0; maximumDepthInMeters: 25; locationRemarks: Long term observatory; verbatimLatitude: 45 3 42.66N; verbatimLongitude: 13d 32' 56.976'' E; verbatimSRS: WGS84; coordinatePrecision: 0.00001

#### Euduboscquella crenulata

Swift ex Elbrächter & Drebes, 1978

30C11F48-178C-539B-92DC-8B8D6C7AEFD7

##### Materials

- **Type status:** Other material. **Occurrence:** individualCount: 11; occurrenceID: 575FA6C6-CA9C-5A38-9BB7-7B7382B3ACC3; **Location:** waterBody: Adriatic Sea; country: Croatia; locality: RV004; verbatimDepth: 0-25 m; minimumDepthInMeters: 0; maximumDepthInMeters: 25; locationRemarks: Long term observatory; verbatimLatitude: 45 3 42.66N; verbatimLongitude: 13d 32' 56.976'' E; verbatimSRS: WGS84; coordinatePrecision: 0.00001

#### Goniodoma polyedricum

Kamptner, 1943

B7419541-89AF-50F5-B219-B8397F1F8AF7

##### Materials

- **Type status:** Other material. **Occurrence:** individualCount: 24; occurrenceID: B7F1837B-1D0A-5FE7-B640-A30AFD4D8E1A; **Location:** waterBody: Adriatic Sea; country: Croatia; locality: RV001; verbatimDepth: 0-25 m; minimumDepthInMeters: 0; maximumDepthInMeters: 25; locationRemarks: Long term observatory; verbatimLatitude: 45 4 48N; verbatimLongitude: 13d 36' 36'' E; verbatimSRS: WGS84; coordinatePrecision: 0.00001
- **Type status:** Other material. **Occurrence:** individualCount: 7; occurrenceID: 760DAA87-1190-547C-8C95-12C68C0F03C4; **Location:** waterBody: Adriatic Sea; country: Croatia; locality: RV004; verbatimDepth: 0-25 m; minimumDepthInMeters: 0; maximumDepthInMeters: 25; locationRemarks: Long term observatory; verbatimLatitude: 45 3 42.66N; verbatimLongitude: 13d 32' 56.976'' E; verbatimSRS: WGS84; coordinatePrecision: 0.00001

#### Bacillariophyceae

04D9CFCE-4997-5A98-B373-C292E3B941A2

##### Materials

- **Type status:** Other material. **Occurrence:** individualCount: 27; occurrenceID: 467627C5-BE1E-5102-9896-19EF1C6D6F50; **Location:** waterBody: Adriatic Sea; country: Croatia; locality: RV001; verbatimDepth: 0-25 m; minimumDepthInMeters: 0; maximumDepthInMeters: 25; locationRemarks: Long term observatory; verbatimLatitude: 45 4 48N; verbatimLongitude: 13d 36' 36'' E; verbatimSRS: WGS84; coordinatePrecision: 0.00001
- **Type status:** Other material. **Occurrence:** individualCount: 4; occurrenceID: DECB0F03-59B9-53D7-BD13-7D7371FD0F51; **Location:** waterBody: Adriatic Sea; country: Croatia; locality: RV004; verbatimDepth: 0-25 m; minimumDepthInMeters: 0; maximumDepthInMeters: 25; locationRemarks: Long term observatory; verbatimLatitude: 45 3 42.66N; verbatimLongitude: 13d 32' 56.976'' E; verbatimSRS: WGS84; coordinatePrecision: 0.00001

##### Notes

The sequences listed under this taxon were classified into Bacillariophyceae; however, no reference sequence with high enough similarity was available for determining the genus of the respective sequences.

#### Psammodictyon constrictum

(Gregory) D.G.Mann, 1990

2B5B80FE-696B-5AB2-A863-73775D3A17AD

##### Materials

- **Type status:** Other material. **Occurrence:** individualCount: 3; occurrenceID: E81D690F-39C4-5618-AA58-6CBF0258FB97; **Location:** waterBody: Adriatic Sea; country: Croatia; locality: RV001; verbatimDepth: 0-25 m; minimumDepthInMeters: 0; maximumDepthInMeters: 25; locationRemarks: Long term observatory; verbatimLatitude: 45 4 48N; verbatimLongitude: 13d 36' 36'' E; verbatimSRS: WGS84; coordinatePrecision: 0.00001
- **Type status:** Other material. **Occurrence:** individualCount: 3; occurrenceID: 00E81C83-0255-5E0E-9578-DD9DDCAE661A; **Location:** waterBody: Adriatic Sea; country: Croatia; locality: RV004; verbatimDepth: 0-25 m; minimumDepthInMeters: 0; maximumDepthInMeters: 25; locationRemarks: Long term observatory; verbatimLatitude: 45 3 42.66N; verbatimLongitude: 13d 32' 56.976'' E; verbatimSRS: WGS84; coordinatePrecision: 0.00001

#### Pseudo-nitzschia pseudodelicatissima

(Hasle) Hasle, 1993

2272CB1D-2AF9-5F39-B003-F6D1054D38E1

##### Materials

- **Type status:** Other material. **Occurrence:** individualCount: 678; occurrenceID: 66CC5270-95E8-5802-ABF3-108993F9760A; **Location:** waterBody: Adriatic Sea; country: Croatia; locality: RV001; verbatimDepth: 0-25 m; minimumDepthInMeters: 0; maximumDepthInMeters: 25; locationRemarks: Long term observatory; verbatimLatitude: 45 4 48N; verbatimLongitude: 13d 36' 36'' E; verbatimSRS: WGS84; coordinatePrecision: 0.00001
- **Type status:** Other material. **Occurrence:** individualCount: 500; occurrenceID: E029F5EC-80FC-57AA-81F5-1C0AAE85FC6D; **Location:** waterBody: Adriatic Sea; country: Croatia; locality: RV004; verbatimDepth: 0-25 m; minimumDepthInMeters: 0; maximumDepthInMeters: 25; locationRemarks: Long term observatory; verbatimLatitude: 45 3 42.66N; verbatimLongitude: 13d 32' 56.976'' E; verbatimSRS: WGS84; coordinatePrecision: 0.00001

#### Pseudo-nitzschia sp.

17F394B6-5874-52AC-8D8C-A6A9377BF70F

##### Materials

- **Type status:** Other material. **Occurrence:** individualCount: 68; occurrenceID: 3B03ECEA-F0A6-5EAE-9BCB-FF78EEB53ECB; **Location:** waterBody: Adriatic Sea; country: Croatia; locality: RV001; verbatimDepth: 0-25 m; minimumDepthInMeters: 0; maximumDepthInMeters: 25; locationRemarks: Long term observatory; verbatimLatitude: 45 4 48N; verbatimLongitude: 13d 36' 36'' E; verbatimSRS: WGS84; coordinatePrecision: 0.00001
- **Type status:** Other material. **Occurrence:** individualCount: 47; occurrenceID: 221D4852-9908-5152-AA4A-366741C81026; **Location:** waterBody: Adriatic Sea; country: Croatia; locality: RV004; verbatimDepth: 0-25 m; minimumDepthInMeters: 0; maximumDepthInMeters: 25; locationRemarks: Long term observatory; verbatimLatitude: 45 3 42.66N; verbatimLongitude: 13d 32' 56.976'' E; verbatimSRS: WGS84; coordinatePrecision: 0.00001

#### Bacteriastrum hyalinum

Lauder, 1864

0E8BD71C-0FA1-5D86-91C3-4E8C06F5FC26

##### Materials

- **Type status:** Other material. **Occurrence:** individualCount: 11; occurrenceID: F88B5AE3-5038-5CB6-A5E9-79AEC19DAB54; **Location:** waterBody: Adriatic Sea; country: Croatia; locality: RV001; verbatimDepth: 0-25 m; minimumDepthInMeters: 0; maximumDepthInMeters: 25; locationRemarks: Long term observatory; verbatimLatitude: 45 4 48N; verbatimLongitude: 13d 36' 36'' E; verbatimSRS: WGS84; coordinatePrecision: 0.00001
- **Type status:** Other material. **Occurrence:** individualCount: 1; occurrenceID: F9BF9790-5CC3-5979-848B-50EC4B6167CE; **Location:** waterBody: Adriatic Sea; country: Croatia; locality: RV004; verbatimDepth: 0-25 m; minimumDepthInMeters: 0; maximumDepthInMeters: 25; locationRemarks: Long term observatory; verbatimLatitude: 45 3 42.66N; verbatimLongitude: 13d 32' 56.976'' E; verbatimSRS: WGS84; coordinatePrecision: 0.00001

#### Bacteriastrum jadranum

Godrijan, Maric & Pfannkuchen, 2012

806E68C6-A1A0-5B68-8A1A-EE2912716E99

##### Materials

- **Type status:** Other material. **Occurrence:** individualCount: 2; occurrenceID: 718C3E91-C492-5544-985F-B3A8973D1BDC; **Location:** waterBody: Adriatic Sea; country: Croatia; locality: RV004; verbatimDepth: 0-25 m; minimumDepthInMeters: 0; maximumDepthInMeters: 25; locationRemarks: Long term observatory; verbatimLatitude: 45 3 42.66N; verbatimLongitude: 13d 32' 56.976'' E; verbatimSRS: WGS84; coordinatePrecision: 0.00001

#### Chaetoceros anastomosans

Grunow, 1882

67D7B33A-1A5B-5731-A8D3-A0765B732ED4

##### Materials

- **Type status:** Other material. **Occurrence:** individualCount: 4; occurrenceID: C61CE334-510A-5312-9B33-F0ABD216807B; **Location:** waterBody: Adriatic Sea; country: Croatia; locality: RV001; verbatimDepth: 0-25 m; minimumDepthInMeters: 0; maximumDepthInMeters: 25; locationRemarks: Long term observatory; verbatimLatitude: 45 4 48N; verbatimLongitude: 13d 36' 36'' E; verbatimSRS: WGS84; coordinatePrecision: 0.00001
- **Type status:** Other material. **Occurrence:** individualCount: 10; occurrenceID: 941F6CE5-E908-523E-A5D1-ED8019271073; **Location:** waterBody: Adriatic Sea; country: Croatia; locality: RV004; verbatimDepth: 0-25 m; minimumDepthInMeters: 0; maximumDepthInMeters: 25; locationRemarks: Long term observatory; verbatimLatitude: 45 3 42.66N; verbatimLongitude: 13d 32' 56.976'' E; verbatimSRS: WGS84; coordinatePrecision: 0.00001

#### Chaetoceros brevis

F.Schütt, 1895

EF47CC1B-7537-52A4-B268-3D7109D88E6B

##### Materials

- **Type status:** Other material. **Occurrence:** individualCount: 7; occurrenceID: 9A7B7387-4E9F-5DC5-8598-B78D4B23E52F; **Location:** waterBody: Adriatic Sea; country: Croatia; locality: RV001; verbatimDepth: 0-25 m; minimumDepthInMeters: 0; maximumDepthInMeters: 25; locationRemarks: Long term observatory; verbatimLatitude: 45 4 48N; verbatimLongitude: 13d 36' 36'' E; verbatimSRS: WGS84; coordinatePrecision: 0.00001
- **Type status:** Other material. **Occurrence:** individualCount: 2; occurrenceID: A7A65519-F58F-5CB3-9484-074563257B8E; **Location:** waterBody: Adriatic Sea; country: Croatia; locality: RV004; verbatimDepth: 0-25 m; minimumDepthInMeters: 0; maximumDepthInMeters: 25; locationRemarks: Long term observatory; verbatimLatitude: 45 3 42.66N; verbatimLongitude: 13d 32' 56.976'' E; verbatimSRS: WGS84; coordinatePrecision: 0.00001

#### Chaetoceros costatus

Pavillard, 1911

A497C436-804D-51B4-895E-5E1247704C57

##### Materials

- **Type status:** Other material. **Occurrence:** individualCount: 16; occurrenceID: B6DE37D4-3E46-5D50-9382-5FA5206F1F46; **Location:** waterBody: Adriatic Sea; country: Croatia; locality: RV001; verbatimDepth: 0-25 m; minimumDepthInMeters: 0; maximumDepthInMeters: 25; locationRemarks: Long term observatory; verbatimLatitude: 45 4 48N; verbatimLongitude: 13d 36' 36'' E; verbatimSRS: WGS84; coordinatePrecision: 0.00001

#### Chaetoceros curvisetus

Cleve, 1889

F54E4E16-ABC4-50BE-AC9B-A9F8B88586E1

##### Materials

- **Type status:** Other material. **Occurrence:** individualCount: 10; occurrenceID: 517F08E5-21A4-558B-91FC-D901927B1C53; **Location:** waterBody: Adriatic Sea; country: Croatia; locality: RV001; verbatimDepth: 0-25 m; minimumDepthInMeters: 0; maximumDepthInMeters: 25; locationRemarks: Long term observatory; verbatimLatitude: 45 4 48N; verbatimLongitude: 13d 36' 36'' E; verbatimSRS: WGS84; coordinatePrecision: 0.00001
- **Type status:** Other material. **Occurrence:** individualCount: 5; occurrenceID: C8305D3E-47E0-5B92-B6BC-DCABF5C74056; **Location:** waterBody: Adriatic Sea; country: Croatia; locality: RV004; verbatimDepth: 0-25 m; minimumDepthInMeters: 0; maximumDepthInMeters: 25; locationRemarks: Long term observatory; verbatimLatitude: 45 3 42.66N; verbatimLongitude: 13d 32' 56.976'' E; verbatimSRS: WGS84; coordinatePrecision: 0.00001

#### Chaetoceros danicus

Cleve, 1889

49A0634F-FEE0-550F-896D-D764E0084D44

##### Materials

- **Type status:** Other material. **Occurrence:** individualCount: 8; occurrenceID: 6649B7E1-8F97-51BA-A57B-36EA3C0267C5; **Location:** waterBody: Adriatic Sea; country: Croatia; locality: RV001; verbatimDepth: 0-25 m; minimumDepthInMeters: 0; maximumDepthInMeters: 25; locationRemarks: Long term observatory; verbatimLatitude: 45 4 48N; verbatimLongitude: 13d 36' 36'' E; verbatimSRS: WGS84; coordinatePrecision: 0.00001
- **Type status:** Other material. **Occurrence:** individualCount: 5; occurrenceID: 21CA35DC-7845-5857-BFF9-7B200A720A56; **Location:** waterBody: Adriatic Sea; country: Croatia; locality: RV004; verbatimDepth: 0-25 m; minimumDepthInMeters: 0; maximumDepthInMeters: 25; locationRemarks: Long term observatory; verbatimLatitude: 45 3 42.66N; verbatimLongitude: 13d 32' 56.976'' E; verbatimSRS: WGS84; coordinatePrecision: 0.00001

#### Chaetoceros decipiens

Cleve, 1873

B3357186-721E-5872-AB96-97A990A7CA70

##### Materials

- **Type status:** Other material. **Occurrence:** individualCount: 17; occurrenceID: 4420E984-2D98-564F-BBC3-14DCE3D74615; **Location:** waterBody: Adriatic Sea; country: Croatia; locality: RV001; verbatimDepth: 0-25 m; minimumDepthInMeters: 0; maximumDepthInMeters: 25; locationRemarks: Long term observatory; verbatimLatitude: 45 4 48N; verbatimLongitude: 13d 36' 36'' E; verbatimSRS: WGS84; coordinatePrecision: 0.00001

#### Chaetoceros diversus

Cleve, 1873

C8F23897-C16A-58AD-880B-8F6DB73838B7

##### Materials

- **Type status:** Other material. **Occurrence:** individualCount: 2; occurrenceID: 25AB51F4-247E-5A65-B485-1A5578B712AF; **Location:** waterBody: Adriatic Sea; country: Croatia; locality: RV001; verbatimDepth: 0-25 m; minimumDepthInMeters: 0; maximumDepthInMeters: 25; locationRemarks: Long term observatory; verbatimLatitude: 45 4 48N; verbatimLongitude: 13d 36' 36'' E; verbatimSRS: WGS84; coordinatePrecision: 0.00001

#### Chaetoceros eibenii

Grunow, 1882

3E4023FB-C64D-5D7F-B3F3-48A9B20BCE6B

##### Materials

- **Type status:** Other material. **Occurrence:** individualCount: 2; occurrenceID: 1DFFE39F-CEE1-529D-BAF5-2DE2970D7D77; **Location:** waterBody: Adriatic Sea; country: Croatia; locality: RV001; verbatimDepth: 0-25 m; minimumDepthInMeters: 0; maximumDepthInMeters: 25; locationRemarks: Long term observatory; verbatimLatitude: 45 4 48N; verbatimLongitude: 13d 36' 36'' E; verbatimSRS: WGS84; coordinatePrecision: 0.00001
- **Type status:** Other material. **Occurrence:** individualCount: 1; occurrenceID: 954071BB-D4B0-5C34-B231-8F2E8422CF70; **Location:** waterBody: Adriatic Sea; country: Croatia; locality: RV004; verbatimDepth: 0-25 m; minimumDepthInMeters: 0; maximumDepthInMeters: 25; locationRemarks: Long term observatory; verbatimLatitude: 45 3 42.66N; verbatimLongitude: 13d 32' 56.976'' E; verbatimSRS: WGS84; coordinatePrecision: 0.00001

#### Chaetoceros lauderi

Ralfs, 1864

4872DC20-C5BB-5A33-8780-52F6FC81A25F

##### Materials

- **Type status:** Other material. **Occurrence:** individualCount: 25; occurrenceID: AEDE31CD-8D0F-5A0E-94C0-A520616D6192; **Location:** waterBody: Adriatic Sea; country: Croatia; locality: RV001; verbatimDepth: 0-25 m; minimumDepthInMeters: 0; maximumDepthInMeters: 25; locationRemarks: Long term observatory; verbatimLatitude: 45 4 48N; verbatimLongitude: 13d 36' 36'' E; verbatimSRS: WGS84; coordinatePrecision: 0.00001
- **Type status:** Other material. **Occurrence:** individualCount: 19; occurrenceID: B5812D8F-1752-5658-AFB2-076EA0572C5E; **Location:** waterBody: Adriatic Sea; country: Croatia; locality: RV004; verbatimDepth: 0-25 m; minimumDepthInMeters: 0; maximumDepthInMeters: 25; locationRemarks: Long term observatory; verbatimLatitude: 45 3 42.66N; verbatimLongitude: 13d 32' 56.976'' E; verbatimSRS: WGS84; coordinatePrecision: 0.00001

#### Chaetoceros socialis

H.S.Lauder, 1864

B2B66134-32B8-58A8-917E-90C35CEE59E3

##### Materials

- **Type status:** Other material. **Occurrence:** individualCount: 76; occurrenceID: E2A6602A-B651-5E18-A394-06B378E3904F; **Location:** waterBody: Adriatic Sea; country: Croatia; locality: RV001; verbatimDepth: 0-25 m; minimumDepthInMeters: 0; maximumDepthInMeters: 25; locationRemarks: Long term observatory; verbatimLatitude: 45 4 48N; verbatimLongitude: 13d 36' 36'' E; verbatimSRS: WGS84; coordinatePrecision: 0.00001
- **Type status:** Other material. **Occurrence:** individualCount: 9; occurrenceID: F9ABC834-93F7-53F1-BFEA-A2D212BE050A; **Location:** waterBody: Adriatic Sea; country: Croatia; locality: RV004; verbatimDepth: 0-25 m; minimumDepthInMeters: 0; maximumDepthInMeters: 25; locationRemarks: Long term observatory; verbatimLatitude: 45 3 42.66N; verbatimLongitude: 13d 32' 56.976'' E; verbatimSRS: WGS84; coordinatePrecision: 0.00001

#### Chaetoceros sp.

E4C60DCE-2A6B-5CDF-98D3-E0BB11EEF159

##### Materials

- **Type status:** Other material. **Occurrence:** individualCount: 20; occurrenceID: 712E90A2-9678-5025-A44A-2607FD083F3C; **Location:** waterBody: Adriatic Sea; country: Croatia; locality: RV001; verbatimDepth: 0-25 m; minimumDepthInMeters: 0; maximumDepthInMeters: 25; locationRemarks: Long term observatory; verbatimLatitude: 45 4 48N; verbatimLongitude: 13d 36' 36'' E; verbatimSRS: WGS84; coordinatePrecision: 0.00001
- **Type status:** Other material. **Occurrence:** individualCount: 191; occurrenceID: A51650D9-FB4F-5330-9AC9-34C060DD8AEB; **Location:** waterBody: Adriatic Sea; country: Croatia; locality: RV004; verbatimDepth: 0-25 m; minimumDepthInMeters: 0; maximumDepthInMeters: 25; locationRemarks: Long term observatory; verbatimLatitude: 45 3 42.66N; verbatimLongitude: 13d 32' 56.976'' E; verbatimSRS: WGS84; coordinatePrecision: 0.00001

#### Chaetoceros tenuissimus

Meunier, 1913

4BCD2D68-12B3-5233-9A95-E39669807DC9

##### Materials

- **Type status:** Other material. **Occurrence:** individualCount: 17; occurrenceID: B2484546-F129-5375-9C9B-A845CA5C2585; **Location:** waterBody: Adriatic Sea; country: Croatia; locality: RV004; verbatimDepth: 0-25 m; minimumDepthInMeters: 0; maximumDepthInMeters: 25; locationRemarks: Long term observatory; verbatimLatitude: 45 3 42.66N; verbatimLongitude: 13d 32' 56.976'' E; verbatimSRS: WGS84; coordinatePrecision: 0.00001

#### Coscinodiscus wailesii

Gran & Angst, 1931

167B10FB-98E0-5B07-8EBE-2C7A6AEF0ED0

##### Materials

- **Type status:** Other material. **Occurrence:** individualCount: 4; occurrenceID: 343AFE8E-DF32-519D-950D-7B6018B6B0AD; **Location:** waterBody: Adriatic Sea; country: Croatia; locality: RV001; verbatimDepth: 0-25 m; minimumDepthInMeters: 0; maximumDepthInMeters: 25; locationRemarks: Long term observatory; verbatimLatitude: 45 4 48N; verbatimLongitude: 13d 36' 36'' E; verbatimSRS: WGS84; coordinatePrecision: 0.00001
- **Type status:** Other material. **Occurrence:** individualCount: 5; occurrenceID: 5F91517A-2443-56AA-B82D-8CB6B3C07D62; **Location:** waterBody: Adriatic Sea; country: Croatia; locality: RV004; verbatimDepth: 0-25 m; minimumDepthInMeters: 0; maximumDepthInMeters: 25; locationRemarks: Long term observatory; verbatimLatitude: 45 3 42.66N; verbatimLongitude: 13d 32' 56.976'' E; verbatimSRS: WGS84; coordinatePrecision: 0.00001

#### Papiliocellulus elegans

Hasle, Stosch & Syvertsen, 1983

FE721F2B-9158-5AD5-A6D6-D7713075B19C

##### Materials

- **Type status:** Other material. **Occurrence:** individualCount: 360; occurrenceID: 982A7196-335F-5FE3-88CB-EF38412C5936; **Location:** waterBody: Adriatic Sea; country: Croatia; locality: RV001; verbatimDepth: 0-25 m; minimumDepthInMeters: 0; maximumDepthInMeters: 25; locationRemarks: Long term observatory; verbatimLatitude: 45 4 48N; verbatimLongitude: 13d 36' 36'' E; verbatimSRS: WGS84; coordinatePrecision: 0.00001
- **Type status:** Other material. **Occurrence:** individualCount: 72; occurrenceID: 2AEC1AB5-E7D4-5A8A-88B3-3D52A7F8A4AC; **Location:** waterBody: Adriatic Sea; country: Croatia; locality: RV004; verbatimDepth: 0-25 m; minimumDepthInMeters: 0; maximumDepthInMeters: 25; locationRemarks: Long term observatory; verbatimLatitude: 45 3 42.66N; verbatimLongitude: 13d 32' 56.976'' E; verbatimSRS: WGS84; coordinatePrecision: 0.00001

#### Bellerochea polymorpha

(Hargraves & Guillard) Hasle, Stosch & Syvertsen, 1983

CCB6AE13-D9A9-52C1-8D18-DA7483AE730B

##### Materials

- **Type status:** Other material. **Occurrence:** individualCount: 9; occurrenceID: 09936AF8-3E03-51F5-BFFF-8B8DB2AF39A9; **Location:** waterBody: Adriatic Sea; country: Croatia; locality: RV001; verbatimDepth: 0-25 m; minimumDepthInMeters: 0; maximumDepthInMeters: 25; locationRemarks: Long term observatory; verbatimLatitude: 45 4 48N; verbatimLongitude: 13d 36' 36'' E; verbatimSRS: WGS84; coordinatePrecision: 0.00001

#### Cerataulina bergonii

(Cleve) Hendey, 1937

DD15AF8E-3C49-5F07-AA0F-3B86343B9D77

##### Materials

- **Type status:** Other material. **Occurrence:** individualCount: 57; occurrenceID: 41009D08-1F62-5651-B218-0F2C3E7A30C1; **Location:** waterBody: Adriatic Sea; country: Croatia; locality: RV001; verbatimDepth: 0-25 m; minimumDepthInMeters: 0; maximumDepthInMeters: 25; locationRemarks: Long term observatory; verbatimLatitude: 45 4 48N; verbatimLongitude: 13d 36' 36'' E; verbatimSRS: WGS84; coordinatePrecision: 0.00001
- **Type status:** Other material. **Occurrence:** individualCount: 91; occurrenceID: 4E23E3FA-ED6F-562C-B670-329DFDF44391; **Location:** waterBody: Adriatic Sea; country: Croatia; locality: RV004; verbatimDepth: 0-25 m; minimumDepthInMeters: 0; maximumDepthInMeters: 25; locationRemarks: Long term observatory; verbatimLatitude: 45 3 42.66N; verbatimLongitude: 13d 32' 56.976'' E; verbatimSRS: WGS84; coordinatePrecision: 0.00001

#### Hemiaulus chinensis

Greville, 1865

C6FB0B63-A24F-538D-85D0-280B5C7387BA

##### Materials

- **Type status:** Other material. **Occurrence:** individualCount: 9; occurrenceID: C173CA07-813D-59B3-8A28-AF402038DE3A; **Location:** waterBody: Adriatic Sea; country: Croatia; locality: RV001; verbatimDepth: 0-25 m; minimumDepthInMeters: 0; maximumDepthInMeters: 25; locationRemarks: Long term observatory; verbatimLatitude: 45 4 48N; verbatimLongitude: 13d 36' 36'' E; verbatimSRS: WGS84; coordinatePrecision: 0.00001
- **Type status:** Other material. **Occurrence:** individualCount: 6; occurrenceID: 918DD70E-2772-5D25-A34E-66A4B5C6A5E0; **Location:** waterBody: Adriatic Sea; country: Croatia; locality: RV004; verbatimDepth: 0-25 m; minimumDepthInMeters: 0; maximumDepthInMeters: 25; locationRemarks: Long term observatory; verbatimLatitude: 45 3 42.66N; verbatimLongitude: 13d 32' 56.976'' E; verbatimSRS: WGS84; coordinatePrecision: 0.00001

#### Cymbella prostrata

(Berkeley) Grun, 1880

DF3C337A-F8D7-565F-B582-C2802DC3953F

##### Materials

- **Type status:** Other material. **Occurrence:** individualCount: 17; occurrenceID: 0E77E73E-C654-576D-BF33-81D57A8FF104; **Location:** waterBody: Adriatic Sea; country: Croatia; locality: RV001; verbatimDepth: 0-25 m; minimumDepthInMeters: 0; maximumDepthInMeters: 25; locationRemarks: Long term observatory; verbatimLatitude: 45 4 48N; verbatimLongitude: 13d 36' 36'' E; verbatimSRS: WGS84; coordinatePrecision: 0.00001

#### Leptocylindrus aporus

(F.W.French & Hargraves) D.Nanjappa & A.Zingone, 2013

AD8ACCB4-EB52-59C1-8295-34326029D03D

##### Materials

- **Type status:** Other material. **Occurrence:** individualCount: 3; occurrenceID: 2316B36A-9497-5F37-A0A1-6E03ACB98DF1; **Location:** waterBody: Adriatic Sea; country: Croatia; locality: RV004; verbatimDepth: 0-25 m; minimumDepthInMeters: 0; maximumDepthInMeters: 25; locationRemarks: Long term observatory; verbatimLatitude: 45 3 42.66N; verbatimLongitude: 13d 32' 56.976'' E; verbatimSRS: WGS84; coordinatePrecision: 0.00001

#### Leptocylindrus convexus

D.Nanjappa & A.Zingone, 2013

9A698791-DB2D-5F4B-98CD-BDB96F37B58F

##### Materials

- **Type status:** Other material. **Occurrence:** individualCount: 1; occurrenceID: 1BF4FEC8-F0B9-5F41-B6A6-D9809115E322; **Location:** waterBody: Adriatic Sea; country: Croatia; locality: RV001; verbatimDepth: 0-25 m; minimumDepthInMeters: 0; maximumDepthInMeters: 25; locationRemarks: Long term observatory; verbatimLatitude: 45 4 48N; verbatimLongitude: 13d 36' 36'' E; verbatimSRS: WGS84; coordinatePrecision: 0.00001
- **Type status:** Other material. **Occurrence:** individualCount: 4; occurrenceID: EAEE999F-6082-5C4F-8F1E-98E0C606210C; **Location:** waterBody: Adriatic Sea; country: Croatia; locality: RV004; verbatimDepth: 0-25 m; minimumDepthInMeters: 0; maximumDepthInMeters: 25; locationRemarks: Long term observatory; verbatimLatitude: 45 3 42.66N; verbatimLongitude: 13d 32' 56.976'' E; verbatimSRS: WGS84; coordinatePrecision: 0.00001

#### Leptocylindrus danicus

Cleve, 1889

F37EADCF-E533-52EF-A59A-01406A2B057C

##### Materials

- **Type status:** Other material. **Occurrence:** individualCount: 17; occurrenceID: C995071C-326E-5986-B060-494D062A632C; **Location:** waterBody: Adriatic Sea; country: Croatia; locality: RV004; verbatimDepth: 0-25 m; minimumDepthInMeters: 0; maximumDepthInMeters: 25; locationRemarks: Long term observatory; verbatimLatitude: 45 3 42.66N; verbatimLongitude: 13d 32' 56.976'' E; verbatimSRS: WGS84; coordinatePrecision: 0.00001

#### Meuniera membranacea

(Cleve) P.C.Silva, 1996

9160AFFD-989F-55B5-9CCF-D7804C7E0049

##### Materials

- **Type status:** Other material. **Occurrence:** individualCount: 1; occurrenceID: D55927C8-4765-5389-B3ED-F1E0E9F17E7C; **Location:** waterBody: Adriatic Sea; country: Croatia; locality: RV001; verbatimDepth: 0-25 m; minimumDepthInMeters: 0; maximumDepthInMeters: 25; locationRemarks: Long term observatory; verbatimLatitude: 45 4 48N; verbatimLongitude: 13d 36' 36'' E; verbatimSRS: WGS84; coordinatePrecision: 0.00001

#### Navicula sp.

99FF7D40-BD15-590B-8FA1-F7ACFA141FC2

##### Materials

- **Type status:** Other material. **Occurrence:** individualCount: 7; occurrenceID: 152E4CF4-45F9-538E-8426-7506B76D771A; **Location:** waterBody: Adriatic Sea; country: Croatia; locality: RV001; verbatimDepth: 0-25 m; minimumDepthInMeters: 0; maximumDepthInMeters: 25; locationRemarks: Long term observatory; verbatimLatitude: 45 4 48N; verbatimLongitude: 13d 36' 36'' E; verbatimSRS: WGS84; coordinatePrecision: 0.00001

#### Pinnularia appendiculata

(C.Agardh) Schaarschmidt, 1881

06E6858A-6471-5F5F-AE81-5FBE9EF5CD85

##### Materials

- **Type status:** Other material. **Occurrence:** individualCount: 1; occurrenceID: 89F093AC-B409-5726-91FF-F42245ABEAC2; **Location:** waterBody: Adriatic Sea; country: Croatia; locality: RV001; verbatimDepth: 0-25 m; minimumDepthInMeters: 0; maximumDepthInMeters: 25; locationRemarks: Long term observatory; verbatimLatitude: 45 4 48N; verbatimLongitude: 13d 36' 36'' E; verbatimSRS: WGS84; coordinatePrecision: 0.00001

#### Pleurosigma intermedium

W.Smith, 1853

B08FB0EB-E1A8-5A48-80F7-AA8FF013297D

##### Materials

- **Type status:** Other material. **Occurrence:** individualCount: 1; occurrenceID: 6A3AD67A-1FFD-5F6E-B001-D4F2D03084F7; **Location:** waterBody: Adriatic Sea; country: Croatia; locality: RV001; verbatimDepth: 0-25 m; minimumDepthInMeters: 0; maximumDepthInMeters: 25; locationRemarks: Long term observatory; verbatimLatitude: 45 4 48N; verbatimLongitude: 13d 36' 36'' E; verbatimSRS: WGS84; coordinatePrecision: 0.00001
- **Type status:** Other material. **Occurrence:** individualCount: 2; occurrenceID: 3E2FFBE4-CB8A-5FDC-BB7C-8183C79D5482; **Location:** waterBody: Adriatic Sea; country: Croatia; locality: RV004; verbatimDepth: 0-25 m; minimumDepthInMeters: 0; maximumDepthInMeters: 25; locationRemarks: Long term observatory; verbatimLatitude: 45 3 42.66N; verbatimLongitude: 13d 32' 56.976'' E; verbatimSRS: WGS84; coordinatePrecision: 0.00001

#### Pleurosigma sp.

0F7DB2E4-114C-5FA8-9906-C035A4A9FCD2

##### Materials

- **Type status:** Other material. **Occurrence:** individualCount: 4; occurrenceID: CC88587F-7FD7-5D4E-BCDA-347998589089; **Location:** waterBody: Adriatic Sea; country: Croatia; locality: RV001; verbatimDepth: 0-25 m; minimumDepthInMeters: 0; maximumDepthInMeters: 25; locationRemarks: Long term observatory; verbatimLatitude: 45 4 48N; verbatimLongitude: 13d 36' 36'' E; verbatimSRS: WGS84; coordinatePrecision: 0.00001

#### Sellaphoraceae

2A8E0850-B4F8-5D72-A8F3-6D7DDAC09462

##### Materials

- **Type status:** Other material. **Occurrence:** individualCount: 1; occurrenceID: CE6A50DC-CF75-5255-ACC4-21871D0F4FB9; **Location:** waterBody: Adriatic Sea; country: Croatia; locality: RV001; verbatimDepth: 0-25 m; minimumDepthInMeters: 0; maximumDepthInMeters: 25; locationRemarks: Long term observatory; verbatimLatitude: 45 4 48N; verbatimLongitude: 13d 36' 36'' E; verbatimSRS: WGS84; coordinatePrecision: 0.00001
- **Type status:** Other material. **Occurrence:** individualCount: 3; occurrenceID: EB3FBA9F-2EB2-52D1-893F-651675A64C05; **Location:** waterBody: Adriatic Sea; country: Croatia; locality: RV004; verbatimDepth: 0-25 m; minimumDepthInMeters: 0; maximumDepthInMeters: 25; locationRemarks: Long term observatory; verbatimLatitude: 45 3 42.66N; verbatimLongitude: 13d 32' 56.976'' E; verbatimSRS: WGS84; coordinatePrecision: 0.00001

##### Notes

The sequences listed under this taxon were classified into Sellaphoraceae; however, no reference sequence with high enough similarity was available for determining the genus of the respective sequences.

#### Guinardia flaccida

H. Peragallo, 1892

770467E6-FCA9-5A09-9AEB-29FA6B59A00F

##### Materials

- **Type status:** Other material. **Occurrence:** individualCount: 16; occurrenceID: 953EC524-A10F-57D5-8FE2-07FBFC189CE0; **Location:** waterBody: Adriatic Sea; country: Croatia; locality: RV001; verbatimDepth: 0-25 m; minimumDepthInMeters: 0; maximumDepthInMeters: 25; locationRemarks: Long term observatory; verbatimLatitude: 45 4 48N; verbatimLongitude: 13d 36' 36'' E; verbatimSRS: WGS84; coordinatePrecision: 0.00001
- **Type status:** Other material. **Occurrence:** individualCount: 5; occurrenceID: FDA8D9BA-1258-5A71-82FF-6404CE929AD7; **Location:** waterBody: Adriatic Sea; country: Croatia; locality: RV004; verbatimDepth: 0-25 m; minimumDepthInMeters: 0; maximumDepthInMeters: 25; locationRemarks: Long term observatory; verbatimLatitude: 45 3 42.66N; verbatimLongitude: 13d 32' 56.976'' E; verbatimSRS: WGS84; coordinatePrecision: 0.00001

#### Guinardia sp.

82E88BB6-0CC1-5544-A53A-3EE2F32722F9

##### Materials

- **Type status:** Other material. **Occurrence:** individualCount: 4; occurrenceID: BC150C14-A03A-5486-B12A-A64693D5871E; **Location:** waterBody: Adriatic Sea; country: Croatia; locality: RV001; verbatimDepth: 0-25 m; minimumDepthInMeters: 0; maximumDepthInMeters: 25; locationRemarks: Long term observatory; verbatimLatitude: 45 4 48N; verbatimLongitude: 13d 36' 36'' E; verbatimSRS: WGS84; coordinatePrecision: 0.00001
- **Type status:** Other material. **Occurrence:** individualCount: 21; occurrenceID: 13687DCF-44F5-5BF6-B272-CC772D3A05FC; **Location:** waterBody: Adriatic Sea; country: Croatia; locality: RV004; verbatimDepth: 0-25 m; minimumDepthInMeters: 0; maximumDepthInMeters: 25; locationRemarks: Long term observatory; verbatimLatitude: 45 3 42.66N; verbatimLongitude: 13d 32' 56.976'' E; verbatimSRS: WGS84; coordinatePrecision: 0.00001

#### Proboscia sp.

A459FA3B-1805-5D12-A212-3402DD35788F

##### Materials

- **Type status:** Other material. **Occurrence:** individualCount: 13; occurrenceID: 8622B007-8ABA-5AF9-841F-E5B665FE2FB9; **Location:** waterBody: Adriatic Sea; country: Croatia; locality: RV001; verbatimDepth: 0-25 m; minimumDepthInMeters: 0; maximumDepthInMeters: 25; locationRemarks: Long term observatory; verbatimLatitude: 45 4 48N; verbatimLongitude: 13d 36' 36'' E; verbatimSRS: WGS84; coordinatePrecision: 0.00001
- **Type status:** Other material. **Occurrence:** individualCount: 5; occurrenceID: 2DB48CA6-89A5-551E-B4B5-A2B21F45652A; **Location:** waterBody: Adriatic Sea; country: Croatia; locality: RV004; verbatimDepth: 0-25 m; minimumDepthInMeters: 0; maximumDepthInMeters: 25; locationRemarks: Long term observatory; verbatimLatitude: 45 3 42.66N; verbatimLongitude: 13d 32' 56.976'' E; verbatimSRS: WGS84; coordinatePrecision: 0.00001

#### Pseudosolenia calcar-avis

(Schultze) B.G.Sundström, 1986

5A8C8A21-8C99-5523-971D-5ACCCB40295D

##### Materials

- **Type status:** Other material. **Occurrence:** individualCount: 4; occurrenceID: 3CF855E3-5C2C-5FAD-8EBB-874F0449A54B; **Location:** waterBody: Adriatic Sea; country: Croatia; locality: RV001; verbatimDepth: 0-25 m; minimumDepthInMeters: 0; maximumDepthInMeters: 25; locationRemarks: Long term observatory; verbatimLatitude: 45 4 48N; verbatimLongitude: 13d 36' 36'' E; verbatimSRS: WGS84; coordinatePrecision: 0.00001

#### Rhizosolenia formosa

H.Peragallo, 1888

67EEE3D5-0F3E-5D20-AC05-2E6479A42D7E

##### Materials

- **Type status:** Other material. **Occurrence:** individualCount: 1; occurrenceID: 3EAAF343-0CAF-562E-8000-9728EC15ADF3; **Location:** waterBody: Adriatic Sea; country: Croatia; locality: RV001; verbatimDepth: 0-25 m; minimumDepthInMeters: 0; maximumDepthInMeters: 25; locationRemarks: Long term observatory; verbatimLatitude: 45 4 48N; verbatimLongitude: 13d 36' 36'' E; verbatimSRS: WGS84; coordinatePrecision: 0.00001

#### Lauderia annulata

Cleve, 1873

DF0FCAFF-5670-5D1E-842B-01C2EDCAED16

##### Materials

- **Type status:** Other material. **Occurrence:** individualCount: 3; occurrenceID: 43390A2B-EE31-5663-8A29-F285C09D1115; **Location:** waterBody: Adriatic Sea; country: Croatia; locality: RV001; verbatimDepth: 0-25 m; minimumDepthInMeters: 0; maximumDepthInMeters: 25; locationRemarks: Long term observatory; verbatimLatitude: 45 4 48N; verbatimLongitude: 13d 36' 36'' E; verbatimSRS: WGS84; coordinatePrecision: 0.00001

#### Skeletonema pseudocostatum

L.K.Medlin, 1991

81C67C5F-CEC5-5B00-809E-CC14B7E96B7F

##### Materials

- **Type status:** Other material. **Occurrence:** individualCount: 1; occurrenceID: 28F1CE8C-7CFB-5DDF-A330-7C66F3769239; **Location:** waterBody: Adriatic Sea; country: Croatia; locality: RV001; verbatimDepth: 0-25 m; minimumDepthInMeters: 0; maximumDepthInMeters: 25; locationRemarks: Long term observatory; verbatimLatitude: 45 4 48N; verbatimLongitude: 13d 36' 36'' E; verbatimSRS: WGS84; coordinatePrecision: 0.00001

#### Cyclotella choctawhatcheeana

Prasad, 1990

76D5C46E-103C-541A-A5D5-FA80D356D135

##### Materials

- **Type status:** Other material. **Occurrence:** individualCount: 1; occurrenceID: 4636C09C-28F1-5625-8AD8-E5EFCC61E755; **Location:** waterBody: Adriatic Sea; country: Croatia; locality: RV004; verbatimDepth: 0-25 m; minimumDepthInMeters: 0; maximumDepthInMeters: 25; locationRemarks: Long term observatory; verbatimLatitude: 45 3 42.66N; verbatimLongitude: 13d 32' 56.976'' E; verbatimSRS: WGS84; coordinatePrecision: 0.00001

#### Mediolabrus comicus

(H.Takano) Yang Li 2020

A2CE098C-8753-5C13-9F0A-0F355068717F

##### Materials

- **Type status:** Other material. **Occurrence:** individualCount: 10; occurrenceID: 36C6672E-9718-5BAB-ACB8-421E08E6A37F; **Location:** waterBody: Adriatic Sea; country: Croatia; locality: RV001; verbatimDepth: 0-25 m; minimumDepthInMeters: 0; maximumDepthInMeters: 25; locationRemarks: Long term observatory; verbatimLatitude: 45 4 48N; verbatimLongitude: 13d 36' 36'' E; verbatimSRS: WGS84; coordinatePrecision: 0.00001
- **Type status:** Other material. **Occurrence:** individualCount: 3; occurrenceID: 62557E82-D8FA-542A-A777-06EC69639A02; **Location:** waterBody: Adriatic Sea; country: Croatia; locality: RV004; verbatimDepth: 0-25 m; minimumDepthInMeters: 0; maximumDepthInMeters: 25; locationRemarks: Long term observatory; verbatimLatitude: 45 3 42.66N; verbatimLongitude: 13d 32' 56.976'' E; verbatimSRS: WGS84; coordinatePrecision: 0.00001

#### Chrysophyceae

90E2A46C-7ED4-5474-AA24-EAC5DF3CC224

##### Materials

- **Type status:** Other material. **Occurrence:** individualCount: 5; occurrenceID: 7A7D8974-F1AA-5578-AE99-7DEA44C59C8C; **Location:** waterBody: Adriatic Sea; country: Croatia; locality: RV004; verbatimDepth: 0-25 m; minimumDepthInMeters: 0; maximumDepthInMeters: 25; locationRemarks: Long term observatory; verbatimLatitude: 45 3 42.66N; verbatimLongitude: 13d 32' 56.976'' E; verbatimSRS: WGS84; coordinatePrecision: 0.00001

##### Notes

The sequences listed under this taxon were classified into Chrysophyceae; however, no reference sequence with high enough similarity was available for determining the genus of the respective sequences.

## Analysis

From 45 samples during two years monthly sampling at two long-term monitoring stations, a total number of 2,396,571 reads (with 789,775 unique reads after filtering and removing gaps, or 21.0%) was obtained. Ultimately, a total of 3,161 ASVs, 1307 99%-OTUs and 240 97%-OTUs were detected on genus or species level, of which 168, 193 and 81 were present with five of more reads per cluster (ASVs*, 97%-OTUs* or 99%-OTUs*). In total, 5.3% of ASVs, 14.8% and 59.5% of 99%- and 97%-OTUs were found with five or more reads, respectively (Table 1).

In terms of genetic diversity, the most present phytoplankton groups were Dinophyceae and Bacillariophyceae independent of the applied clustering method used, while other groups, Chlorophyta, Haptophyta, Cryptophyta and Chrysophyceae were present with low numbers of clustering units, contributing with less than 5% in a total number of either ASVs or OTUs per group (Table 1).

**Figure 2.**
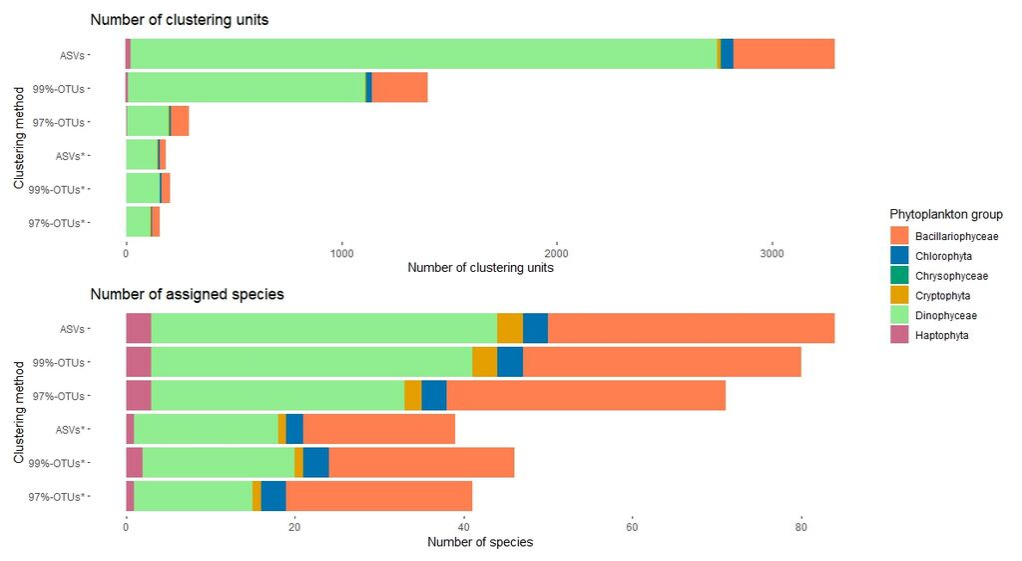
Contribution of phytoplankton groups. Changes in contribution in genetic diversity of different phytoplankton groups depending on clustering method used (upper plot) and number of assigned species of each phytoplankton group depending on clustering method used.

**Figure 3.**
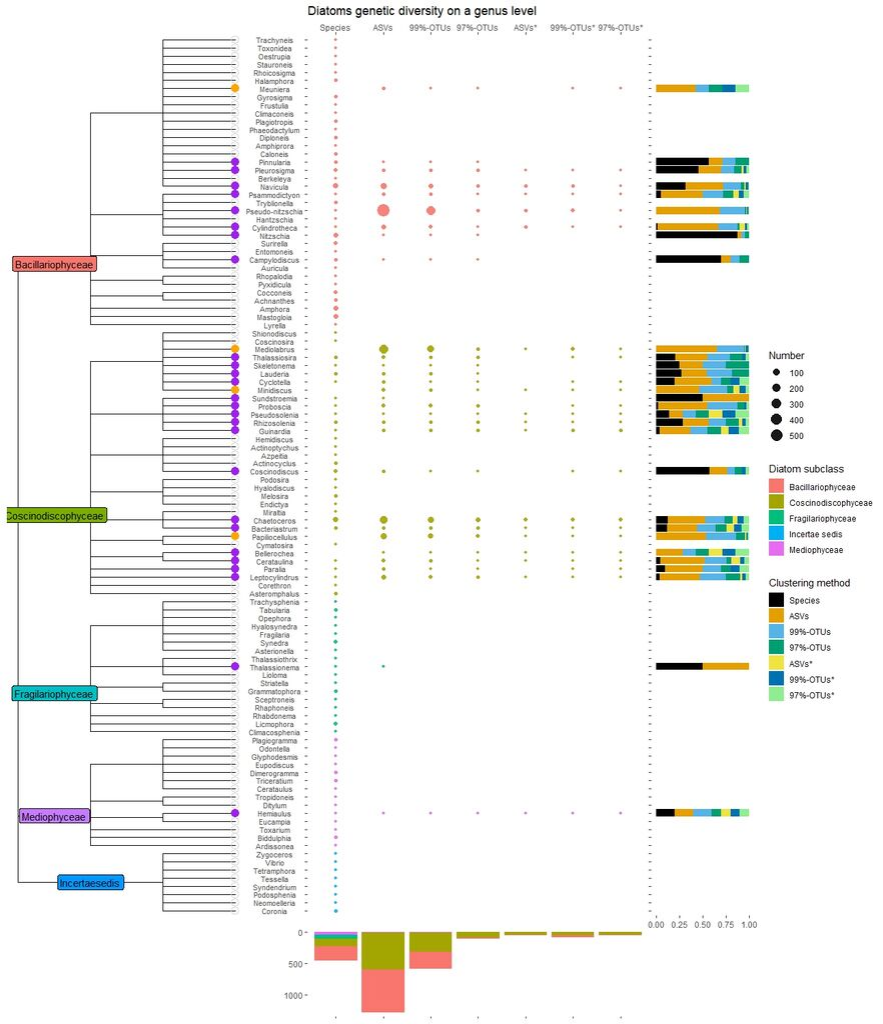
Dendrogram of diatom genera. Dendrogram showing species as listed in Viličić et al. (2002) as recovered from all published and observed data until 2002, representing a potential diatom diversity observable in the eastern northern Adriatic. Genetic diversity acquired in this study is compared to genera found in the checklist (2002). Species recovered from metabarcoding data, but so far absent from the 2002 checklist, were added to the dendrogram: Filled purple dots at the tip points indicate diatom genera obtained with metabarcoding, while orange dots present metabarcoded genera that were not present in a checklist. The bubble plot shows the number of morphospecies from the checklist (column Species) and the number of different clustering units assigned per genera depending on the clustering method (columns ASVs, 99%-OTUs, 97%-OTUs) and removal of low abundant clustering units (columns ASVs*, 99%-OTUs*, 97%-OTUs*). The bar plot on the right showing the contribution of species/clusters for each genus/clustering method. Genera not found in metabarcoding data were left out. The bottom bar plot indicates the number of morphospecies (first column) and clustering units (other columns) generated by each clustering method for the diatom group.

**Figure 4.**
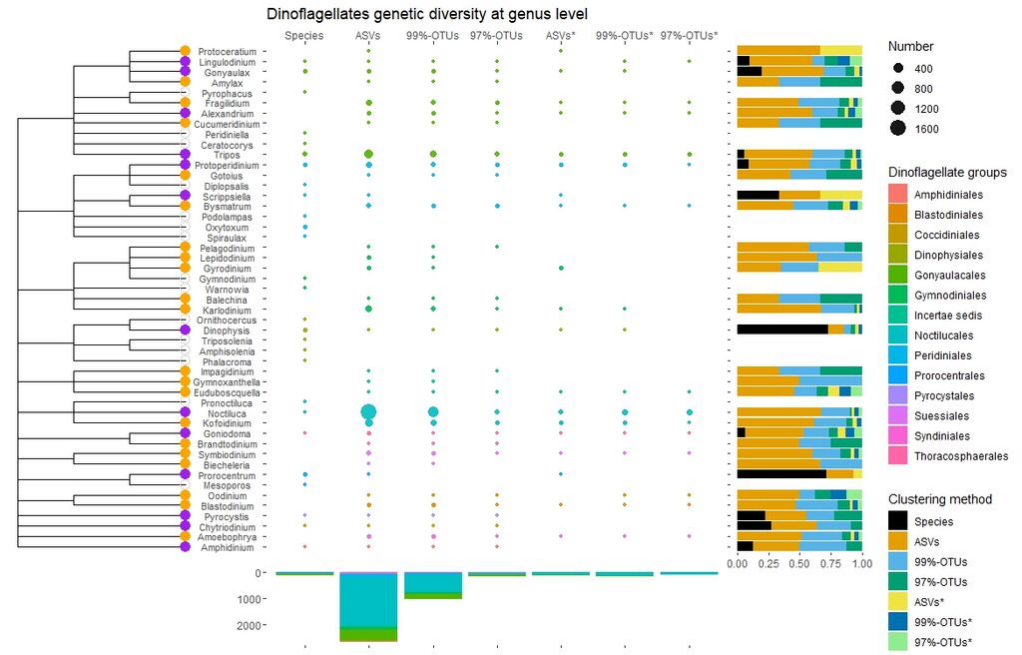
Dendrogram of dinoflagellates genera. Dendrogram showing species as listed in Vilicic et al. (2002) as recovered from all published and observed data until 2002, representing a potential diatom diversity observable in the eastern northern Adriatic. Genetic diversity acquired in this study is compared to genera found in the checklist (2002). Species recovered from metabarcoding data, but so far absent from the 2002 checklist, were added to the dendrogram: Filled purple dots at the tip points indicate diatom genera obtained with metabarcoding, while orange dots present metabarcoded genera that were not present in a checklist. The bubble plot shows the number of morphospecies from the checklist (column Species) and the number of different clustering units assigned per genera depending on the clustering method (columns ASVs, 99%-OTUs, 97%-OTUs) and removal of low abundant clustering units (columns ASVs*, 99%-OTUs*, 97%-OTUs*). The bar plot on the right showing the contribution of species/clusters for each genus/clustering method. Genera not found in metabarcoding data were left out. The bottom bar plot indicates the number of morphospecies (first column) and clustering units (other columns) generated by each clustering method for the dinoflagellate group.

The most diverse group were dinoflagellates, comprising 34 found genera (48.3%) (Fig. 4), followed by diatoms with 23 (35.4%) (Fig. 3) and coccolithophorids with three genera (4.0%). Most taxa were assigned at species level belonging to dinoflagellates, 42 species (or 48.8% of total species) and 36 diatom species (41.7%). Chlorophyta and Haptophyta were represented with three species each (3,5%) and Cryptophyta with two species, both belonging to genus *Teleaulax* (Fig. 2).

As expected, different clustering methods, tend to show different taxonomic coverage. As a result, 84 taxa were assigned at a species level, based on ASV clustering, while with OTU clustering retrieved 80 species (99%-OTUs) and 71 species (97%-OTUs). Using an additional restriction step which relates to the number of reads generated for each ASV or OTU and keeping only those clustering units represented in five or more reads reduced significantly the number of assigned species. A total number of species generated were 39 assigning ASVs*, 46 99%-OTU and 41 regarding 97%-OTU* which correspond to 46.2%, 57.5% and 57.8% of ASVs, 97%-OTUs and 99%-OTUs retained after removing low abundant sequences/clustering units.

The most diverse genera belong to diatoms and dinoflagellates (Suppl. material 1). Diatom genera with the highest number of assigned species were *Chaetoceros* (11) and *Leptocylindrus* (3), along with *Protoperidinium* (5), *Tripos* (5) and *Alexandrium* (3) classified amongst dinoflagellates. Amongst them, genera *Chaetoceros*, *Protoperidinium* and *Tripos* were also assigned up to genus level, indicating even greater diversity within.

Four diatom genera were revealed with molecular methods that have not been reported in previously mentioned checklists. All of them were assigned to species level containing at least one species per genus (*Bellerochea*, *Meuniera*, *Mediolabrus*, *Minidiscus)*. Dinoflagellates showed a higher number of non-reported genera (30) and a higher number of assigned species not mentioned in earlier complete checklists of this area (Figs 3, 4).

## Discussion

Molecular assessments facilitate the investigation of eukaryotic biodiversity by producing a large dataset of sequences for target genetic markers. The sensitivity for species detection is not equally distributed across the observable biodiversity range with significant differences between methodologies (e.g. metabarcodes and light microscopy). Hence a combination of methodologies promises to generate deeper and more complete insights into biodiversity (Huo et al. 2020). In this study, total eukaryotic phytoplankton diversity was explored via the metabarcoding of 18S rDNA gene amplicons and the results for different clustering methods were analysed and further compared with lists of species known for the eastern northern Adriatic. A highly quality controlled reference database incorporating sequences from diverse phytoplankton groups is needed to investigate phytoplankton diversity with metabarcoding data. For this purpose, a custom reference database CIMPhy18 was generated to include as many taxonomically annotated barcode sequences as available. Up-to-date curation of such a database is recommended (Rimet et al. 2019).

Applying metabarcoding to 2 years of monthly sampling set (which is, to our knowledge, currently the most extensive sampling regime for a metabarcoding approach to date for the Mediterranean area), we could recover several previously not listed species for the study area. Some of these were unknown during the generation of the previous checklist (Table 2) and newly described in the meantime, but are today considered a common constituent of eastern northern Adriatic plankton diversity (e.g. *Bacteriastrumjadranum* (Godrijan et al. (2012) and *Leptocylindrusaporus* (Kuzat et al. (2022)). Another new observation is the species *Mediolabruscomicus* belonging to genus *Mediolabrus* separated from the genus *Minidiscus* as a new genus (Li et al. 2020). Such observations highlight how molecular approaches can detect cryptic species and serve as important approaches for the (taxonomic) revision of phytoplankton biodiversity (Kermarrec et al. 2013).

Species from groups other than diatoms and dinoflagellates, especially Chlorophyta, Chrysophyceae and Cryptophyta, have been poorly reported earlier, probably due to their cell size, belonging to nano- and picoplankton fractions which results in difficulties to morphologically determine them at species or genus level.

Larger species, like *Noctilucascintillans*, significantly contributed to the overall read numbers, 49.0% of all generated ASVs were assigned to *Noctiluca* genus. This is an effect of extended and massive blooms of *N.scintilans* during our sampling period. These blooms are known to drastically reduce species diversity in a given area. Even though we enlarged the checklist for the phytoplankton of the eastern Adriatic coast with the here-reported results, we assume for methodological reasons that a part of the observable biodiversity still remains unknown. Several genera known as predominant benthic genera that are highly represented in phytoplankton checklists, such as *Mastogloia*, *Licmophora* or *Synedra* (Car et al. 2021), but are less likely to be found in a water column, were not represented in our metabarcoding results, which is probably a result of the sampling methodology, where we did avoid net hauls close to the sea floor. As described earlier, variable DNA extraction efficiencies between taxa due to different cell morphologies, for example, robust cell coverings, such as the diatom silica frustule, could potentially lessen extraction efficiency, leaving them undetected in metabarcoding efforts (Maki et al. 2017). It is shown that various clustering methods potentially have an impact on community structure, either by means of the proportion for different groups contributing to the total community or, more noticeably, altering taxonomic coverage of a given community. Genetic community composition discrepancy between ASVs and OTUs as the basis for the taxonomic ranking has been previously described (Jeske and Gallert 2022). Assigning taxonomy to different OTUs which represent a consensus sequence at the respective centroid of the cluster and, therefore, closely-related species (such as species from *Pseudo-nitzschia* complex) could be overcome using oppressing clustering conditions leading to an underestimation of phytoplankton diversity in the environment. On the other hand, ASVs as a denoising method appear more desirable lately (Callahan et al. 2017). With one nucleotide tolerance, the probability for assigning genotypes due to sequencing errors is reduced (Kelly et al. 2019) allowing unconstrained identification of closely-related species or intraspecific genetic diversity detection. Consequently, diversity estimates including OTUs or ASVs, not assigned to known species, have to be interpreted with restraint. We hence suggest that our results at the level of genus can be used for an estimation of intrageneric diversity potentially recoverable from the observed environment (Figs 3, 4). However, further research, including the analysis of intrageneric and intraspecific phylogenetic distances for the applied barcode, is necessary to confidently predict the biodiversity at the species level for genera with low numbers of available reference barcodes.

By disregarding sequences or sequence clusters constructed, based on low abundant reads (ASVs*, 99%-OTU* and 97%-OTU*), we attempted the elimination of potentially remaining erroneous sequences. Removing those sequences can negatively affect describing community compositions and greatly reduce the number of taxa present in extremely low copy number due to their reduced amplification ability or low abundance in the community structure. It is shown that ASV methods control errors sufficiently down to the level of single-nucleotide differences over the sequenced gene region and, therefore, we assumed that these low abundant sequences are a product of genetic diversity and not a laboratory bias and that even low read number sequences and sequence clusters should be taken into account when creating species lists.

The metabarcoding technique is a promising tool for revealing phytoplankton communities, although gaps in the reference databases still exist (Weigand et al. 2019). To check CIMPhy18 database completeness, we compared it to published checklists of phytoplankton for the study area. In total, 20 phytoplankton genera known from the area (Vilicic et al. 2002) (eight Bacillariophyceae, four Dinophyceae and eight Prymnesiophyceae) could not be found in our custom database nor in any publicly available database. Depending on a reference database completeness, sequence gaps as such result in reduced detection power.

Reference checklists for the study area always include observations for the entire eastern Adriatic coast. Not necessarily all species are present in the northern Adriatic Sea. Furthermore, the checklists integrate over several decades, while the here-presented metabarcode analysis integrates over 2 years of sampling only.

Nevertheless, the here-reported and characterised results from a 2-year, monthly metabarcode analysis of phytoplankton net hauls resulted in the observation of genetic diversity of not only species already known from the studied area, but could also add additional, so far not reported species for the north-eastern Adriatic Sea. Metabarcoding in combination with light and electron microscopy techniques can significantly improve the assessment of the phytoplankton community (Ruppert et al. 2019, Huo et al. 2020, Pereira et al. 2021) and deliver deeper insights into planktonic biodiversity.

## Supplementary Material

5ED9AB99-6498-5B75-BA08-4F9617547011 10.3897/BDJ.11.e106947.suppl1 Supplementary material 1 List of taxa retrived in this study, during 2 years monthly sampling Data type taxa list Brief description List of taxa retrieved in this study, during 2 years monthly sampling. Taxonomic identification according to sequences retrieved from metabarcodes of the samples. File: oo_890889.xls https://binary.pensoft.net/file/890889 Lana Grižančić, Ana Baričević, Mirta Smodlaka Tanković, Ivan Vlašiček, Mia Knjaz, Ivan Podolšak, Tjaša Kogovšek, Martin Pfannkuchen, Daniela Marić Pfannkuchen

## Figures and Tables

**Table 1.** Contribution to genetic diversity for each phytoplankton group and clustering method used

Phytoplankton group	ASVs (%)	99%-OTUs (%)	97%-OTUs (%)	ASVs* (%)	99%-OTUs* (%)	97%-OTUs* (%)
Dinophyceae	82.8	78.6	65.3	77.4	74.5	47.0
Bacillariophyceae	14.3	18.2	27.8	16.1	18.6	39.8
Chlorophyta	1.7	1.9	2.4	4.8	4.4	7.2
Haptophyta	0.6	0.7	2.4	0.5	1.5	3.6
Cryptophyta	0.5	0.4	1.4	1.1	1.0	2.4
Chrysophyceae	0.2	0.2	0.7	0.0	0.0	0.0

**Table 2.** Phytoplankton species revealed by metabarcoding, assigning taxonomy, based on ASVs as a clustering method with no reads cutoff, not present in checklist (Vilicic et al. 2002)

Phytoplankton group	Order	Genus	Species	Author
Bacillariophyceae	Chaetocerotales	*Bacteriastrum*	*Bacteriastrumjadranum*	Godrijan, Maric & Pfannkuchen (2012)
Bacillariophyceae	Chaetocerotales	*Chaetoceros*	*Chaetoceroseibenii*	Grunow (1882)
Bacillariophyceae	Leptocylindrales	*Leptocylindrus*	*Leptocylindrusaporus*	(F.W.French & Hargraves) D.Nanjappa & A.Zingone (2013)
Bacillariophyceae	Thalassiosirales	*Mediolabrus*	*Mediolabruscomicus*	(H.Takano) Yang Li (2020)
Bacillariophyceae	Naviculales	*Meuniera*	*Meunieramembranacea*	P.C.Silva (1996)
Bacillariophyceae	Bacillariales	*Pseudo-nitzschia*	*Pseudo-nitzschiapseudodelicatissima*	(Hasle) Hasle (1993)
Bacillariophyceae	Rhizosoleniales	*Rhizosolenia*	*Rhizosoleniaformosa*	H.Peragallo (1888)
Bacillariophyceae	Thalassiosirales	*Skeletonema*	*Skeletonemapseudocostatum*	L.K.Medlin (1991)
Dinophyceae	Gonyaulacales	*Alexandrium*	*Alexandriumaffine*	(H.Inoue & Y.Fukuyo) Balech (1995)
Dinophyceae	Gonyaulacales	*Alexandrium*	*Alexandriumtamarense*	(Lebour) Balech (1995)
Dinophyceae	Amphidiniales	*Amphidinium*	*Amphidiniummassartii*	Biecheler (1952)
Dinophyceae	Gonyaulacales	*Amylax*	*Amylaxtriacantha*	(Jørgensen) Sournia (1984)
Dinophyceae	Gymnodiniales	*Balechina*	*Balechinagracilis*	(Bergh) F.Gómez, Artigas & Gast (2021)
Dinophyceae	Blastodiniales	*Blastodinium*	*Blastodiniummangini*	Chatton (1908)
Dinophyceae	Blastodiniales	*Blastodinium*	*Blastodiniumspinulosum*	Chatton (1912)
Dinophyceae	Gonyaulacales	*Cucumeridinium*	*Cucumeridiniumcoeruleum*	(Dogiel) F.Gomez, P.López-García, H.Takayama & D.Moreira (2015)
Dinophyceae	Pyrocystales	*Dissodinium*	*Dissodiniumpseudolunula*	Swift ex Elbrächter & Drebes (1978)
Dinophyceae	Gonyaulacales	*Fragilidium*	*Fragilidiummexicanum*	Balech (1988)
Dinophyceae	Gonyaulacales	*Fragilidium*	*Fragilidiumsubglobosum*	(Stosch) Loeblich III (1980)
Dinophyceae	Gonyaulacales	*Gonyaulax*	*Gonyaulaxellegaardiae*	Mertens, Aydin, Takano, Yamaguchi & Matsuoka (2015)
Dinophyceae	Gymnodiniales	*Karlodinium*	*Karlodiniumveneficum*	(D.Ballantine) J.Larsen (2000)
Dinophyceae	Noctilucales	*Kofoidinium*	*Kofoidiniumcf.pavillardii*	J.Cachon & M.Cachon (1967)
Dinophyceae	Gymnodiniales	*Lepidodinium*	*Lepidodiniumchlorophorum*	(M.Elbrächter & E.Schnepf) Gert Hansen, Botes & Salas (2007)
Dinophyceae	Gymnodiniales	*Pelagodinium*	*Pelagodiniumbei*	(H.J.Spero) Siano, Montresor, Probert & Vargas (2010)
Dinophyceae	Dinophysiales	*Phalacroma*	*Phalacromaporodictyum*	Stein (1883)
Dinophyceae	Gonyaulacales	*Protoceratium*	*Protoceratiumreticulatum*	(Claparède & Lachmann) Bütschli (1885)
Chlorophyta	Mamiellales	*Bathycoccus*	*Bathycoccusprasinos*	W.Eikrem & J.Throndsen (1990)
Haptophyta	Isochrysidales	*Gephyrocapsa*	*Gephyrocapsaoceanica*	Kamptner (1943)
Haptophyta	Pavlovales	*Pavlova*	*Pavlovapinguis*	J.C.Green (1967)
Haptophyta	Phaeocystales	*Phaeocystis*	*Phaeocystisglobosa*	Scherffel (1899)
Cryptophyta	Pyrenomonadales	*Teleaulax*	*Teleaulaxamphioxeia*	(W.Conrad) D.R.A.Hill (1992)
Cryptophyta	Pyrenomonadales	*Teleaulax*	*Teleaulaxgracilis*	Laza-Martinez (2012)
Chlorophyta	Chlorodendrales	*Tetraselmis*	*Tetraselmismarina*	(Cienkowski) R.E.Norris, Hori & Chihara (1980)
